# G-quadruplex dynamics contribute to regulation of mitochondrial gene expression

**DOI:** 10.1038/s41598-019-41464-y

**Published:** 2019-04-03

**Authors:** M. Falabella, J. E. Kolesar, C. Wallace, D. de Jesus, L. Sun, Y. V. Taguchi, C. Wang, T. Wang, I. M. Xiang, J. K. Alder, R. Maheshan, W. Horne, J. Turek-Herman, P. J. Pagano, C. M. St. Croix, N. Sondheimer, L. A. Yatsunyk, F. B. Johnson, B. A. Kaufman

**Affiliations:** 1 Vascular Medicine Institute, University of Pittsburgh, Pittsburgh, PA 15261 USA; 2 Department of Animal Biology, University of Pennsylvania, Philadelphia, PA 19104 USA; 3 Center for Biological Imaging, University of Pittsburgh, Pittsburgh, PA 15261 USA; 4 Department of Pediatrics, University of Pittsburgh, Pittsburgh, PA 15224 USA; 5 Department of Chemistry and Biochemistry, Swarthmore College, Swarthmore, PA 19081 USA; 6 Department of Medicine, Division of Pulmonary, Allergy and Critical Care Medicine, University of Pittsburgh, Pittsburgh, PA 15261 USA; 7 Division of Clinical and Metabolic Genetics, The Hospital for Sick Children and the University of Toronto, Toronto, ON M5G 1X8 Canada; 8 Richard K. Mellon Institute for Pediatric Research, Children’s Hospital of Pittsburgh of UPMC, Pittsburgh, PA 15224 USA; 9 Department of Pathology and Laboratory Medicine, University of Pennsylvania Perelman School of Medicine, Philadelphia, PA 19104 USA

## Abstract

Single-stranded DNA or RNA sequences rich in guanine (G) can adopt non-canonical structures known as G-quadruplexes (G4). Mitochondrial DNA (mtDNA) sequences that are predicted to form G4 are enriched on the heavy-strand and have been associated with formation of deletion breakpoints. Increasing evidence supports the ability of mtDNA to form G4 in cancer cells; however, the functional roles of G4 structures in regulating mitochondrial nucleic acid homeostasis in non-cancerous cells remain unclear. Here, we demonstrate by live cell imaging that the G4-ligand RHPS4 localizes primarily to mitochondria at low doses. We find that low doses of RHPS4 do not induce a nuclear DNA damage response but do cause an acute inhibition of mitochondrial transcript elongation, leading to respiratory complex depletion. We also observe that RHPS4 interferes with mtDNA levels or synthesis both in cells and isolated mitochondria. Importantly, a mtDNA variant that increases G4 stability and anti-parallel G4-forming character shows a stronger respiratory defect in response to RHPS4, supporting the conclusion that mitochondrial sensitivity to RHPS4 is G4-mediated. Taken together, our results indicate a direct role for G4 perturbation in mitochondrial genome replication, transcription processivity, and respiratory function in normal cells.

## Introduction

Mitochondria are flexible organelles that play a crucial metabolic role in stress and disease resilience. Mitochondria are bigenomic, with subunits of the multimeric oxidative phosphorylation (OXPHOS) complexes encoded by both nuclear and mitochondrial DNA (mtDNA). Genetic lesions arising in either the Mendelian nuclear genome or the matrilineal multicopy mtDNA can result in mitochondrial dysfunction. Mammalian mtDNA is a circular genome characterized by strand asymmetry with guanine enrichment in the “heavy” strand. During first strand mtDNA replication, the heavy strand is displaced from the replicating light strand^1^, increasing the potential for its damage. Importantly, the primary driver of age-acquired mutations is thought to be cytosine deamination and not guanine oxidation in mtDNA^2,3^. The heavy-strand guanine enrichment may thus reduce the incidence of cytosine to thymidine transitions on this strand and the probability of mutations^4,5^.

One effect of guanine enrichment in the heavy strand is the increased potential for intramolecular G-quadruplex (G4) formation during replication. G-quadruplex structures can form in sequences that have four proximal runs of two or more consecutive guanines. The frequency of sequences with G4-forming potential in mtDNA appears to be non-random^6^, with many potential G4s conserved among species and verified to form G4 structures *in vitro*^6,7^. In cancer cells, there is a strong association between the positions of nuclear genome deletion breakpoints and the sequences predicted to form G4 structures, suggesting that the stalling of replication machinery induced by unresolved G4s is a contributing factor in deletion formation^8^. Our group and others have also shown that potential G4-forming sequences are associated with mtDNA deletion breakpoints found in patients with mitochondrial disorders^6,7,9^.

The association of predicted G4-forming sequences with mtDNA deletions does not prove that G4 structures cause nucleic acid instability *per se*, nor does it define the potential roles that G4s have on biological function. A biological effect of mitochondrial G4 was suggested when we and our collaborators described a subtle increase in age-associated mtDNA deletion burden in the skeletal muscle of mice ablated for the PIF1 G4-helicase^10^. However, the small increase in mtDNA deletions was not commensurate with the notable respiratory complex deficiency in the skeletal muscle of those mice^10^ or in PIF1 knockdown cells^11^. Intriguingly, it has been shown that in *in vitro* mitochondrial transcription assays, hybrid G4 structures are formed at specific sites early in the nascent transcript to cause transcript termination, suggesting that G4s could alter gene expression^12–14^. In mtDNA, there are many other non-randomly distributed and well-conserved sequences with G4-forming potential, which compelled further investigation into their potential biological impact on mitochondrial function.

To this end, we sought to identify approaches that alter mitochondrial G-quadruplex stability and assess the effect on mtDNA metabolism and respiratory function in normal cells^15^. The presence of G4-forming sequences at telomeres and oncogene promoters has made G4 structures a potential target in anticancer therapies^16^. Hence, in the past years a great number of small molecules have been designed to bind and stabilize the G4 structures to damage cancer cells. As such, the development of G4 ligands is a growing field^17^. The large number of G4 binding molecules has been used both as a tool to gain new insights in the G4 biology (e.g. Yang *et al*.^18^) and as potential anticancer agents (e.g. Asamitsu *et al*.^19^). Recently, the small G4 binding molecule 3,6-bis(1-methyl-4-vinylpyridinium) carbazole diiodide (BMVC), has been shown to localize to mitochondria and induce apoptosis in cancer cell lines^20^. Another compound, 3,11-Difluoro-6,8,13-trimethylquino[4,3,2-*kl*]acridinium methylsulfate (RHPS4), was reported to be a G4-selective ligand^21–23^ with nuclear localization^24,25^ that preferentially affected the viability of cancer cell lines (Supplementary Dataset S1). Here, we show that RHPS4 preferentially localizes to mitochondria in non-cancerous mouse embryonic fibroblast cell lines (MEFs) and affects mitochondrial genome content, transcription elongation, and respiratory function. We also find that RHPS4 causes differential effects on mitochondrial function in genome variants associated with alterations in the G4-forming potential. Leveraging the ability of RHPS4 to cause transcription or replication defects depending on concentration, we identify novel gene expression pathways that distinguish between these processes. Together, these results strengthen the notion that mitochondrial G-quadruplex dysregulation affects mitochondrial nucleic acid synthesis and mitochondrial function beyond association with mtDNA deletion formation.

## Results

### Identification of RHPS4 as a mitochondrial G4 ligand that affects mtDNA replication

**Figure 1:**
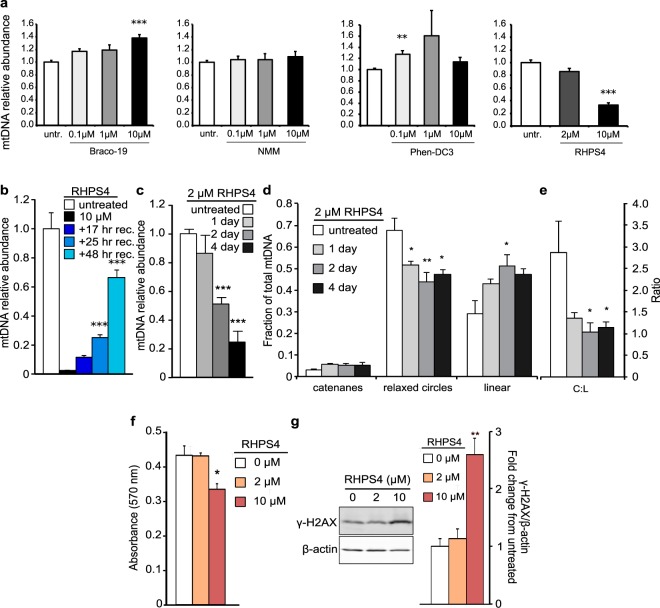
G4-ligand exposure causes reversible mtDNA depletion. (**a**) Determination of mouse embryonic fibroblasts (MEFs) sensitivity to different G-quadruplexes ligands. mtDNA depletion analysed by qPCR in WT MEFs cultured in the presence of Braco-19, NMM, Phen-DC3, or RHPS4 at the indicated concentrations for 24 hours (untr.; untreated). Each treatment set is normalized to untreated control. All data are mean +/− SEM (n = 4; p-values calculated by one-way ANOVA with Dunnett’s posthoc analysis: **< 0.01, ***<0.001). (**b**) Reversibility of mtDNA depletion in WT MEFs. Cells were exposed to 10 µM RHPS4 for 4 days and followed by recovery (rec.) for the indicated time. (**c**) Time-dependent mtDNA depletion as analysed by qPCR. (**d**) Alterations in the relative abundance of mtDNA catenanes, relaxed circles, and linear molecules from panel C time course. (**e**) RHPS4 decreases the mtDNA circular to linear ratio (C:L). (**f**) Crystal violet assay on MEFs treated with RHPS4 for 24 hours. (**g**) Western blot analysis of phospho-γ-H2AX of protein extracts from MEFs treated with RHPS4 for 24 hours. β-actin is shown as the loading control. All data are mean +/− SEM (n = 3–8; p-values calculated by one-way ANOVA with Dunnett’s posthoc analysis: *<0.05, **<0.01, ***<0.001).

Our objective was to stabilize G4 structures in mitochondria to determine their impact on mitochondrial DNA, gene expression and respiratory function. In the nucleus, ligand-mediated G4 stabilization is known to affect DNA replication leading to genome instability^26^. By extension, we expected that small molecules that increase G4 stability in mitochondria would also cause replication defects, leading to mtDNA depletion^15^. We tested four known G4 ligands (Braco-19, NMM, Phen-DC3, and RHPS4) for their ability to induce mtDNA depletion in cultured mouse embryonic fibroblast (MEF) cells (Fig. 1a). Because mitochondrial localization of small molecules has been observed with both neutral and positively charged lipophilic compounds, these compounds were selected as representatives of different scaffold structures or charge. The positively charged compound RHPS4 induced significant and reproducible depletion of mtDNA content. We further established that the RHPS4-mediated decrease of mtDNA levels was reversible (Fig. 1b) and time-dependent (Fig. 1c). Supporting the notion that G4 stabilization would interfere with replication, we found that 2 µM RHPS4 treatment reduced the ratio of circular to linear mtDNA content (Fig. 1d,e; Supplementary Dataset S2), a feature previously observed in dideoxycytosine (ddC) and hydrogen peroxide-induced mtDNA damage and depletion^27^.

We next confirmed that RHPS4 preferentially binds G4 structures vs. double-stranded (ds) DNA, which is crucial to the interpretation of the biological data. Toward that end, F21D, which is a widely used, G4-forming human telomere sequence, was subjected to fluorescence resonance energy transfer (FRET)-melting assays^28^ in the presence of increasing amounts of RHPS4. We found that sub-micromolar RHPS4 concentrations were sufficient to increase the melting temperature of F21D by at least 15 °C (Supplementary Fig. S1a). This stabilization was poorly competed by 480 equivalents of non-specific dsDNA (Supplementary Fig. S1b). These data demonstrate that RHPS4 binding stabilizes G4 structures and confirm the strong selectivity of RHPS4 for G4 structures over dsDNA consistent with previous reports^21–23^.

To exclude that the mtDNA depletion observed at 2 µM RHPS4 was caused by induction of nuclear DNA damage response (DDR), we investigated the effect of RHPS4 (at 2 and 10 µM) on MEFs viability (Fig. 1f), telomere length (Supplementary Fig. S2a) and γ-H2AX abundance; the latter is a well-established marker of nuclear DNA damage^29^ (Fig. 1g, Supplementary Fig. S2b and Supplementary Dataset S3). In cells treated with 2 µM RHPS4 for 24 hrs, we did not observe any effect on cell proliferation, γ-H2AX abundance, or telomere length assessed by fluorescent *in situ* hybridization. On the contrary, 10 µM RHPS4 did not induce telomere shortening (Supplementary Fig. S2a) but had a mild effect on cell proliferation (Fig. 1f) and γ-H2AX abundance (Fig. 1g). We confirmed the increase of γ-H2AX levels by *in situ* imaging (Supplementary Fig. S2b). Taken together, our data demonstrate that RHPS4 at low dose (2 µM) interferes with mtDNA copy number in cells, in the absence of detectable nuclear DDR. To avoid the influence of nuclear DNA damage, we used 2 µM RHPS4 treatment for 24 h to study the effect of G4 stabilization on mitochondrial function.

**Figure 2:**
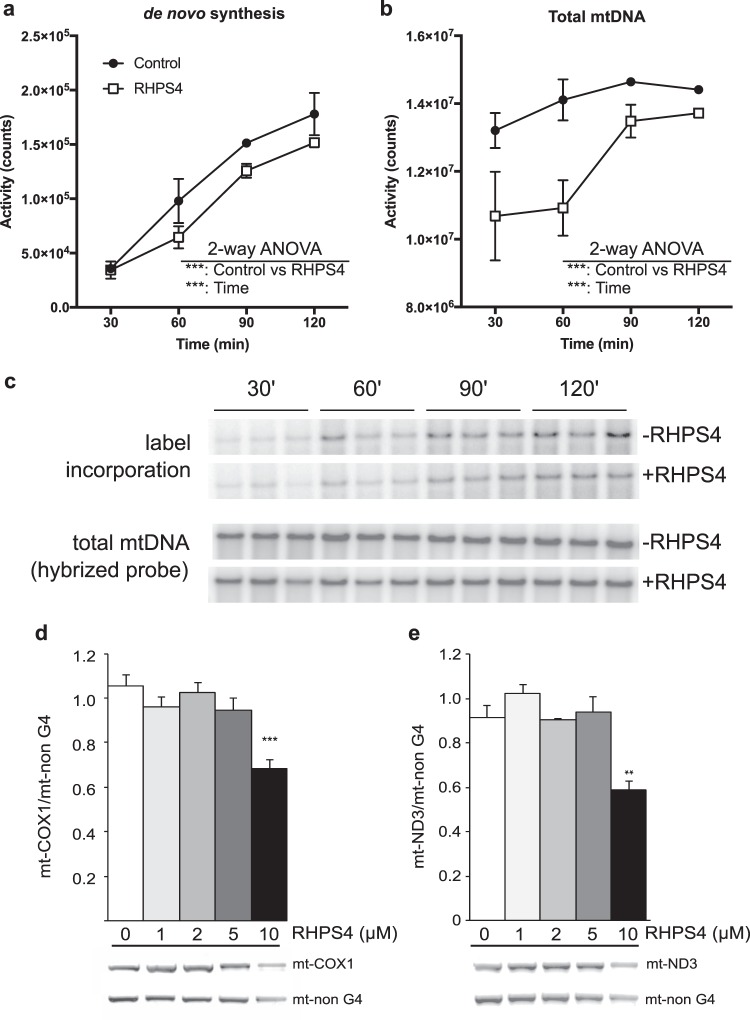
RHPS4 reduces mtDNA replication or polymerization *in vitro*. (**a**,**b**) DNA replication in isolated mouse liver mitochondria in the presence or absence of 2 µM RHPS4. (**a**) Radiolabel incorporation into full-length mtDNA across a 30–120 min time course. (**b**) Total full-length mtDNA determined by hybridization probing and autoradiography. (**c**) Raw radiographic images of full-length mtDNA. (**d**,**e**) Taq-based PCR stop assay using untreated HeLa cellular DNA amplifying mt-COXI (G4 amplicon; **d**) or mt-ND3 (G4 amplicon; **e**) and mt-non G4 (no G4 in amplicon). Amount of RHPS4 in PCR reaction as indicated. Data are shown as mean +/− SEM (n = 3). P-values determined by 2-way (**a**,**b**) or 1-way ANOVA (d, e; *<0.05; **<0.01; ***<0.001).

To further corroborate that nuclear DNA damage was not involved in the mtDNA alterations observed at low dose of RHPS4 (2 µM), we used an *in organello* replication assay^30,31^ on isolated mouse liver mitochondria (Fig. 2). Isolated mitochondria retain the ability to synthesize DNA without any input from the nucleus or cytoplasm. In this system, we observed that 2 µM RHPS4 was sufficient to inhibit the radiolabelled dCTP incorporation into full-length mtDNA (Fig. 2a) as well as prevent the increase of full-length mtDNA over time (Fig. 2b). This experiment demonstrates that RHPS4 is sufficient to inhibit mtDNA synthesis and RHPS4-nuclear DNA interaction is not required to mediate the replication defect.

To determine whether RHPS4 can bind to mtDNA G4 sequences, we next tested mtDNA sequence amplification by a Taq-based PCR stop assay^20^ using total DNA isolated from HeLa cells (Fig. 2d,e). Importantly, this assay showed decreased amplification efficiency at sites that form mtDNA G4 sequences (COX1 and ND3) and a very mild effect on a non-G4 mtDNA sequence. These results support the hypothesis that RHPS4 interferes preferentially with G4 sequences relative to non-G4 forming sequences to block the synthesis of mtDNA. Together, the *in organello* labelling and the PCR stop data strongly suggest that RHPS4 can recognize mitochondrial sequences and does not require nuclear DNA damage to impact mitochondrial nucleic acid synthesis.

### Live cell imaging microscopy reveals RHPS4 localizes primarily into mitochondria

We next incubated MEF cells with increasing concentrations of RHPS4 (1, 2, and 10 µM) overnight and found that nucleolar localization was apparent only after extended exposure of the treated cells to epifluorescence light sources (demonstrated in Supplementary Fig. S3 movie). This result was consistent with the fluorescence localization previously reported in A549 and MCF-7 cells^22^. Our microscopy data obtained using MEF, 143B and HeLa cells suggest that extended confocal light source exposure causes RHPS4 delocalization from mitochondria to nucleoli. We also found that a combination of nucleolar and diffuse cellular localization occurred in MEF cells after exposure to 30 µM RHPS4 for 30 min followed by methanol fixation (Supplementary Fig. S4a), a method previously reported using A549 and MCF-7 cells^24^. To avoid prolonged confocal light source exposure time and prevent nucleolar localization of RHPS4, we rapidly acquired the images using Swept field confocal microscopy. The fast data acquisition of the Swept field method represents a more physiological condition for RHPS4 localization studies.

**Figure 3:**
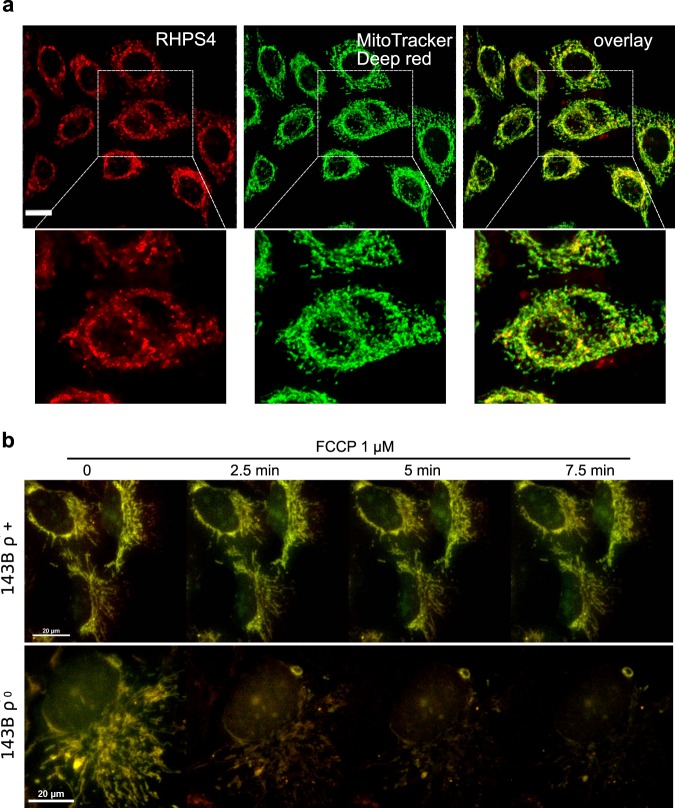
RHPS4 localizes to mitochondria by membrane potential and is retained by binding nucleic acids. (**a**) Single confocal plane images of live MEFs treated with RHPS4 (red) detected in the red channel (λ_ex/em_ = 550/570), co-stained by MitoTracker Deep Red (green), detected in the far red channel (λ_ex/em_ = 640/670) and their merge (yellow) obtained by using high speed confocal microscopy (Nikon Sweptfield). Digital zoom of images shown on right of each panel. Scale bar in panel a is 10 µm. (**b**) Time course of live cell confocal images of 143B ρ^+^ (upper) and 143B ρ^0^ (lower) cells incubated with 1 µM RHPS4 overnight. After the medium was changed, the cells were treated with 1 µM FCCP and multiple individual fields imaged over 15 min. Overlay (yellow) of the endogenous RHPS4 fluorescence detected in the green red (λ_ex/em_ = 470/510) and red (λ_ex/em_ = 550/570) channels. Scale bars in panel b are 20 µm.

To test whether RHPS4 localizes to mitochondria, we performed live cell imaging microscopy using lower doses of RHPS4 compared to the previous studies (Fig. 3). In our hands, overnight exposure to 1 µM RHPS4 was sufficient to achieve fluorescence using 470 nm (green) and 550 nm (red) excitation (emissions at 510 nm and 570 nm, respectively), matching expected spectral characteristics, without signal in the far-red wavelengths. To determine whether the red population localized to mitochondria, we co-stained mitochondria with MitoTracker Deep Red and acquired live cells images in both the red (λ_ex/em_ = 550/570) and far red (λ_ex/em_ = 640/670) channels (Fig. 3a). The RHPS4 fluorescence pattern was not altered by the addition of MitoTracker, and 100% of the RHPS4 volume was positive for MitoTracker Deep Red. Similarly, 96% of the mitochondrial volume (defined by MitoTracker) contained RHPS4. The mitochondrial localization was also confirmed using a commercial source of RHPS4 (Tocris, Bristol, UK, data not shown). Strong mitochondrial localization was observed in three-dimensional renderings of RHPS4 and MitoTracker Deep Red with no discernible nuclear staining in live cells (Supplementary Fig. S5 movie). Unfortunately, attempts to co-localize RHPS4 with mitochondrial sub-compartments using *in situ* immuno co-localization of fixed samples were unsuccessful due to RHPS4 diffusion during processing. Taken together, we provide strong evidence for a mitochondrial localization of RHPS4 at a low dose in live MEFs cells, and we can recapitulate the non-mitochondrial RHPS4 localization by both prolonged fluorescent light source exposure and post-staining fixation.

We next investigated the mechanism of RHPS4 localization to mitochondria. In recent work focused on the study of mitochondrial G4 in cancer cells, the potential G4 ligand BMVC was suggested to localize to mitochondria through the mitochondrial permeability transition pore (PTP)^20^. The PTP is an inner mitochondrial membrane channel known to increase the access of ions and small molecules to the mitochondrial matrix^32^ and it can be blocked by cyclosporin A (CsA). To investigate if RHPS4 also localized to the mitochondrial matrix through the PTP, we incubated the cells with a nontoxic concentration of CsA (1 µM) (Supplementary Fig. S6a) overnight as previously described^20^. To confirm that the CsA treatment blocked the PTP, we used the CoCl_2_-calcein fluorescence-quenching assay^33^. Calcein-AM is a fluorophore able to diffuse to several subcellular compartment, including mitochondria. Upon PTP opening, the cobalt (Co^2+^) enters the mitochondrial matrix and quenches the calcein inducing a rapid decrease of mitochondrial calcein fluorescence. When the PTP is blocked, Co^2+^ is not able to enter the mitochondrial matrix and quench mitochondrial calcein fluorescence. We found that the calcein fluorescence was higher in MEFs pre-treated with CsA, compared to control (Supplementary Fig. S6b,c), confirming the PTP blockage. To further test the effect of CsA pre-treatment, we measured the ΔΨ_m_ using the Tetramethylrhodamine methyl ester (TMRM) method. The TMRM fluorescence was increased in the cells treated with CsA (Supplementary Fig. S6d,e), indicating an increase in mitochondrial membrane potential. After having validated the PTP blockage, we treated the cells with RHPS4 and found that 1 µM CsA pre-treatment increased RHPS4 fluorescence intensity measured in the red channel by live cell imaging (Supplementary Fig. S6f,g). This suggests that RHPS4 localizes to mitochondria without PTP opening and that increased mitochondrial membrane potential facilitates mitochondrial uptake of RHPS4.

We next tested whether mitochondrial membrane potential was essential for RHPS4 uptake. Numerous positively charged compounds localize to mitochondria *via* membrane potential, which has been used to target small molecules to the mitochondrial compartment through moieties such as triphenylphosphonium (TPP)^34^. The targeting of TPP-coupled molecules to mitochondria is blocked by dissipation of the mitochondrial membrane potential. We first preloaded 143B ρ^+^ (normal mtDNA) or ρ^0^ (devoid of mtDNA) cells with 1 µM RHPS4 overnight. After confirming the photostability of the RHPS4 under short repeated measurements (Supplementary Fig. S4b), we followed the effect of the mitochondrial uncoupler FCCP on the RHPS4 localization in live cells (Fig. 3b). We observed a loss of RHPS4 mitochondrial localization after FCCP treatment only in 143B ρ^0^ cells, indicating that membrane potential is sufficient for localization. Because RHPS4 is retained in 143B ρ^+^ cells after FCCP, we suggest an interaction between RHPS4 and mitochondrial nucleic acids. Unfortunately, we were not able to perform an experiment using the reverse exposure order (FCCP then RHPS4) because the cells cannot be maintained in FCCP overnight. Taken together, our data indicates RHPS4 can successfully localize to mitochondria by membrane potential independently of PTP opening.

### RHPS4-mediated respiratory complex depletion occurs through a mitochondrial transcription inhibition mechanism

**Figure 4:**
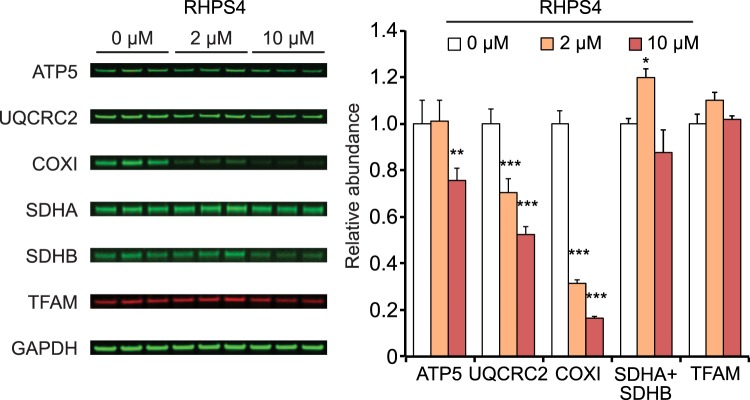
Low-dose RHPS4 exposure causes depletion of representative subunits of complexes III and IV, but not II and V. (Left) Fluorescent western blot analysis of OXPHOS proteins in MEFs cultured in presence 0, 2, and 10 µM RHPS4 for 24 hrs. Transferred membranes were probed for ATP5 (Complex V; CoV), UQCRC2 (Complex III; CoIII), COXI (Complex IV; CoIV), SDHA and SDHB (Complex II; CoII), TFAM, and GAPDH (loading control). Complex I subunits were not detected in MEFs. (Right) Bar graph of OXPHOS proteins and TFAM normalized to GAPDH and control cell exposure levels (0 µM RHPS4). Shown are mean values +/− SEM (n = 3; p-values calculated by one-way ANOVA with Dunnett’s posthoc analysis: *<0.05, **<0.01, ***<0.001).

To determine the impact of RHPS4 exposure on mitochondrial respiratory function, we first examined the abundance of representative subunits of the mitochondrial OXPHOS complexes in MEFs exposed to varying concentrations of RHPS4 (Fig. 4 and Supplementary Dataset S4). MEFs showed significant subunit depletion from Complexes III (UQCRC2) and IV (COX1) at 2 µM RHPS4, whereas the abundance of Complex II subunits (SDHA and SDHB), which are all encoded by the nuclear DNA, and the mitochondrial DNA binding protein TFAM were not affected. We were not able to detect Complex I expression by Western blot in MEF cells. To confirm this observation, we repeated the experiment using murine C2C12 myotubes and found that at 2 µM RHPS4 the abundance of Complex III, IV and I subunits was decreased, but not TFAM or Complex II subunits (Supplementary Fig. S7a). In line with the data obtained in MEF cells (Fig. 1d,e), an increase in the ratio of linear to circular mtDNA forms was also observed in myotubes (Supplementary Fig. S7b,c). These data suggest that RHPS4 effects on mitochondria are general across analysed cell types, not specific to cancer cells as reported in previous studies^35^, and support the hypothesis that low concentrations of RHPS4 induce mitochondrial gene expression defects without affecting the genomic DNA stability.

**Figure 5:**
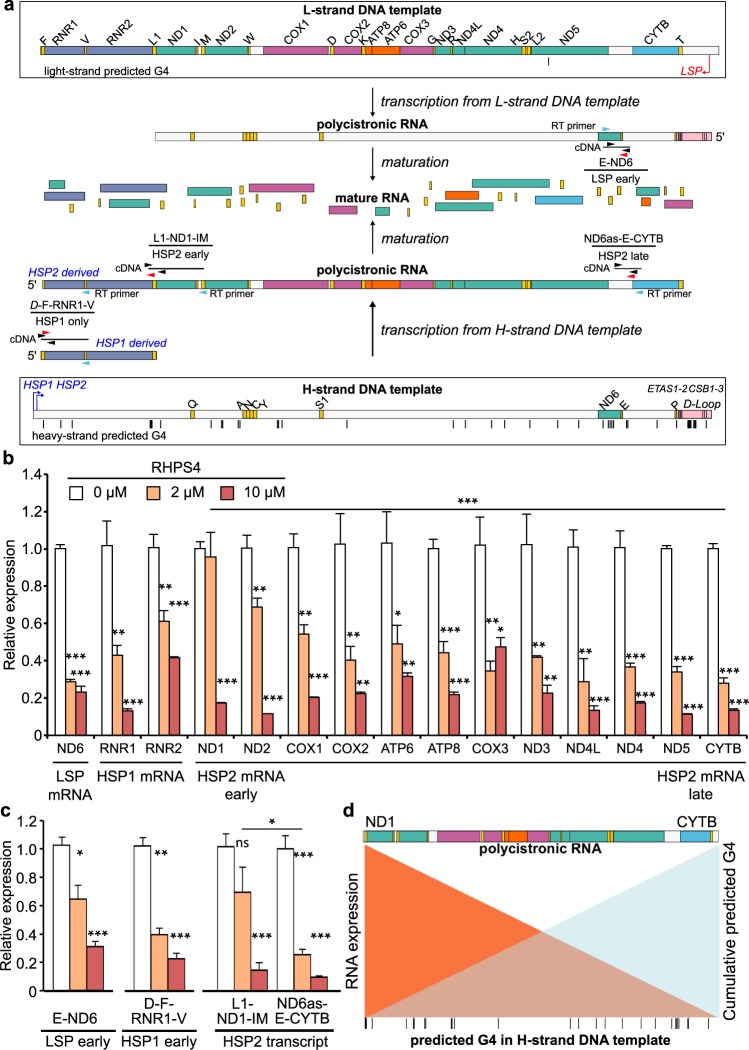
MEF exposure to low-dose RHPS4 causes transcript elongation defects prior to significant mtDNA depletion. (**a**) Schematic representation of mouse mitochondrial polycistronic transcripts and novel specific qRT-PCR assays. Transcription and processing of RNA proceeds from top (L-strand DNA template) or bottom (H-strand DNA template) of panel toward middle as indicated by black arrows. Below the genes there are indicated promoters (Light Strand Promoter, LSP [in red]; Heavy Strand Promoter 1, HSP1 [in blue]; Heavy Strand Promoter 2, HSP2 [in blue]). Transcription from the HSP2 promoter reads the H-strand template DNA (G-rich) to generate L-strand polycistronic RNA (G-poor), proceeding from left to right as a single transcript that is rapidly processed into mRNAs. (**b**) RHPS4 exposure decreases steady-state mature RNA levels assessed by qRT-PCR more extensively with distance from the promoter (HSP2 mRNA late) compared. Each transcript is normalized to GAPDH levels, which were unaffected by RHPS4 exposure. (**c**) HSP2-derived unprocessed transcript assays L-ND1-M and ND6as-E-CYTB confirm elongation defect in the presence of RHPS4. In contrast, LSP early and HSP1 early transcript assays, E-ND6 and D-F-RNR1-V respectively, show potential early transcription interference. (**d**) Model of progressive interference in HSP2-derived unprocessed transcript. All data are mean +/− SEM (n = 3–6; p-values calculated by one-way ANOVA: *<0.05, **<0.01, ***<0.001). Mouse mtDNA sequence was used to predict quadruplex forming potential (QFP; Supplementary Dataset S4) using G4 Hunter^45^.

We next examined alterations in the steady-state transcript levels of mitochondria-encoded genes. Mitochondrial transcription is initiated by three promoters, the Light Strand Promoter (LSP), Heavy Strand Promoter 1 (HSP1), and Heavy Strand Promoter 2 (HSP2; reviewed in^36^). Transcription initiated at these promoters forms polycistronic RNAs that are then efficiently cleaved and processed into mature RNAs (Fig. 5a)^37,38^. Our initial studies used commercial TaqMan probes to detect the relative abundance of the mature RNAs encoded by the two ribosomal and the 13 protein coding genes (non-tRNA RNAs) (Fig. 5b). We noted that LSP and HSP1-derived transcripts (ND6 and RNR1, respectively) were significantly reduced at 2 µM RHPS4 exposure without mtDNA depletion; however, we found that RNAs specific to the HSP2 promoter activity showed greater depletion as distance from HSP2 increased, evidenced from relative effects on ND1 (early transcript) and CYTB (late transcript). The relative abundance of ND6, RNR1, ND1, and CYTB was confirmed by evaluating strand-specific levels of steady-state mitochondrial mRNAs by RNA-Seq (Supplementary Fig. S8). Effects on the HSP2-derived tRNAs loosely recapitulated the promoter distance effect in mRNA; however, the number of sequencing reads was too low for a robust result across all sequences. We also confirmed the RHPS4-mediated effect on ND6, RNR1, ND1 and CYTB by qRT-PCR in cultured C2C12 myotubes (Supplementary Fig. S7d). Using mouse cultured cells, these data indicate that the stabilization of G4 structures alters mitochondrial RNA abundance without having a secondary effect of mtDNA depletion.

Given the decrease in abundance of transcripts with more distance from the HSP2 promoter, we hypothesized that G4s might be interfering with the elongation of transcripts by RNA polymerase. To examine elongation defects in unprocessed mitochondrial RNAs, new assays were required. Standard TaqMan assays are intragenic, detecting both mature and nascent unprocessed RNAs, with unprocessed transcripts typically contributing to a tiny portion of the total mitochondrial RNA^38,39^. To detect the nascent RNA levels, we developed strand-specific intergenic TaqMan assays that measure unprocessed RNAs only (Fig. 5a). Assays for LSP early (E-ND6), HSP1 only (D-F-RNR1-V), and HSP2 early (L1-ND1-IM) showed similar abundance at 2 µM RHPS4 (Fig. 5c) to corresponding mature RNAs (Fig. 5b). As with the mature RNAs, we observed reduced abundance of HSP2 late unprocessed RNA (ND6-as-E-CYTB) relative to HSP2 early sequence (Fig. 5c). The number of potential G4-forming sequences encountered by an elongating polymerase increases with the distance from the promoter, whose cumulative probability would cause a greater decrease in late transcripts. We therefore suggest that the observed HSP2 transcription elongation defect is caused by RHPS4-binding to G4 structures in the template strand because of the limited number of G4 predicted in the RNA (Fig. 5d), but we cannot exclude RHPS4 acting on G4-RNA^18,40^ or potential interactions with other non-canonical structures, such as i-Motifs on the light strand^41^. These alternative RHPS4 targets may contribute to the reduced abundance of ND6 and RNR1/2 mature RNA levels, which are generated by non-HSP2 promoters and appear to be affected by an unprocessed transcript alteration 5′ to those sequences.

Due to the relationship between transcription and replication priming^1^, we also examined potential RHPS4 effects on transcripts associated with replication priming from the RNA-Seq data. Notably, decreased strand-specific RNA abundance at the D-loop (the site of first strand replication initiation) and O_L_ (the site of second strand replication initiation) was detected at 10 µM, but not 2 µM, RHPS4 (Supplementary Fig. S8c), suggesting that the formation of replication-associated transcripts was not impeded at the lower levels of RHPS4, which do not cause mitochondrial genome depletion. We noted that at 10 µM RHPS4 exposure, the distribution of RNA transcripts around CSBI, a typical RNA-DNA transition site, was altered (Supplementary Fig. S8d), suggesting that the robust mtDNA depletion at this high level of RHPS4 could involve effects within the D-loop region. In contrast, the mtDNA damage detected at 2 µM RHPS4 (Fig. 1d,e) apparently occurs independent of direct effects within the D-loop priming RNA.

### Mitochondrial transcription defects induced by RHPS4 are distinct from dsDNA intercalation effects

**Figure 6:**
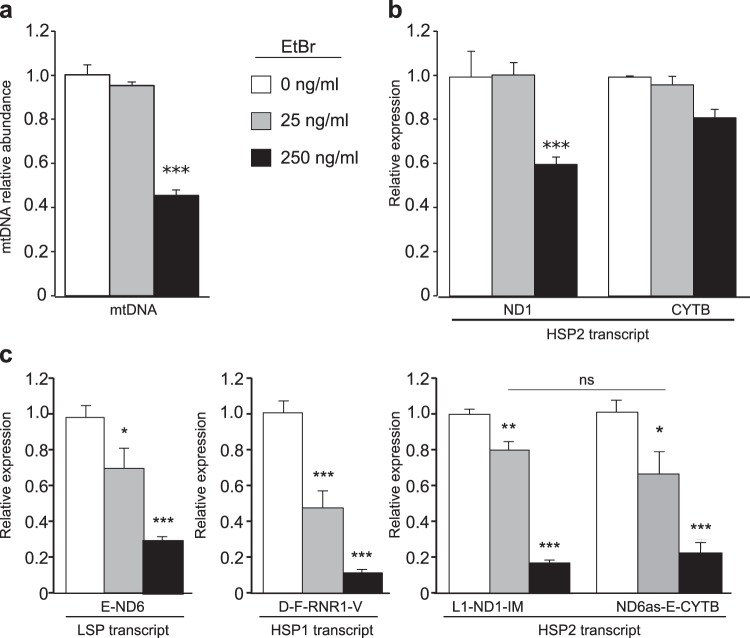
Ethidium bromide treatment does not induce mitochondrial transcription defects prior to mtDNA depletion. (**a**) mtDNA levels were measured in MEF cells treated for 24 hours with 0, 25 and 250 ng/ml EtBr. Concentrations were selected to match mtDNA depletion observed in RHPS4 experiments. (**b**) EtBr treatment does not induce any change in the ND1 and CYTB steady-state transcript levels at concentrations below those that cause mitochondrial genome depletion (25 ng/ml). (**c**) EtBr treatment does not alter parental L-strand transcript ND6 and the H-strand transcript RNR1, ND1 and CYTB levels prior to mtDNA depletion. All data are mean normalized to untreated sample values +/− SEM (n=3; p-values calculated by one-way ANOVA: *<0.05, **<0.01, ***<0.001).

To test the potential for RHPS4-dsDNA interactions as a cause of the observed transcription defect, we tested ethidium bromide (EtBr), a dsDNA intercalating compound that causes mtDNA depletion, for effects on mitochondrial transcription (Fig. 6). We identified EtBr concentrations that recapitulated the extent of mtDNA depletion observed during 2 and 10 µM RHPS4 exposure at 24 hours (Fig. 6a). In contrast to RHPS4, EtBr exposure was only able to cause transcript decrease in the context of mtDNA depletion (Fig. 6b). Notably, the relative abundance of mature CYTB mRNA was not lower than ND1 mRNA (Fig. 6b), there were no differences in the abundance of 5′ and 3′ unprocessed HSP2 sequences (Fig. 6c), and the four unprocessed RNAs from both transcripts were only decreased with mitochondrial genome depletion (Fig. 6c). Thus, at the concentrations tested, EtBr and RHPS4 show distinct effects on mtDNA transcription.

### Differential nuclear transcriptional responses to mitochondrial transcription inhibition and mtDNA depletion

**Figure 7:**
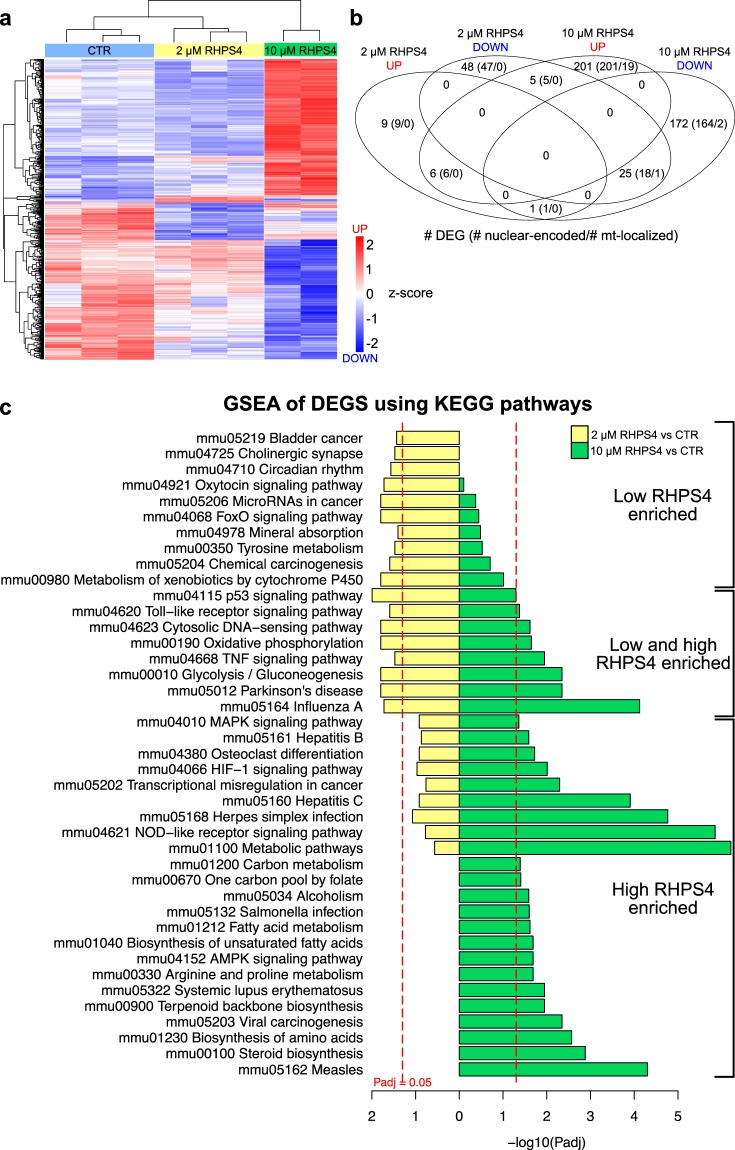
Low and high dose RHPS4 reveals differences in cellular response to mitochondrial transcription inhibition and mtDNA depletion. (**a**) Cluster analysis of differential expressed genes (DEGs) determined by RNA-Seq shows discrete patterns among control (light blue), 2 µM RHPS4 (yellow), and 10 µM RHPS4 (green) treated cells. (**b**) Venn diagram of the number of DEG up regulated (red) or down regulated (blue) in low and high RHPS4 exposure (>1.5 fold, p < 0.01). Subset of DEGs that are nuclear encoded genes and further subset of genes whose products are localized in mitochondria (based on the Mitocarta 2.0 database) are shown in parenthesis. (**c**) Enrichment of DEGs using KEGG pathways. Pathways are separated by low RHPS4 specific, low and high RHPS4 activated, and high RHPS specific changes.

We next examined global cellular transcriptional responses to RHPS4 exposure by RNA-Seq using MEF cells treated with 0, 2, and 10 µM RHPS4 (Fig. 7). Interestingly, the low and high RHPS4 treatment groups showed discrete, differentially expressed genes (DEGs) by cluster analysis (Fig. 7a). A Venn diagram of DEGs, using fold change > 1.5 and adjusted p-value < 0.01 demonstrates the potential for differential effects at the low and high RHPS4 doses (Fig. 7b), as some genes increase expression at one dose and decrease in the other (Supplementary Dataset S5). By analyzing a subset of DEGs for nuclear DNA damage responsive signaling genes (used in Qiagen PAMM-029Z), we did not detect gene expression changes at 2 µM, consistent with our Western blot (Fig. 1g) and *in situ* (Supplementary Fig. S2) results. Only the p53 target genes GADD45A and PPR1R15A were upregulated at 10 µM RHPS4, suggesting a modest nuclear DNA damage effect by exposure to 10 µM RHPS4. These data support the use of 2 µM RHPS4 to study the G4 effects in mitochondria.

Enrichment analysis of the DEGs in KEGG pathways showed specific pathway transitions from low to high dose of RHPS4 (Fig. 7c). The significant pathway alterations support that the DEGs are a response to mitochondrial changes. Of potential relevance to mitochondrial disorders, genes in the oxidative phosphorylation, glycolysis/gluconeogenesis and cytosolic DNA sensing/Toll-like receptor signaling are altered in both RHPS4 conditions. AMPK and HIF1 signaling, steroid biosynthesis, and fatty acid metabolism, among others, showed significant changes in gene expression at the high dose of RHPS4. Interestingly, we detected decreased gene expression affecting serine/glycine, folate, and one-carbon metabolism at 10 µM RHPS4 exposure and concomitant mtDNA depletion, which have been demonstrated to be altered in response to mtDNA replication disorders^42,43^. The molecular determinants of the differential pathway activation between low and high RHPS4 exposure are unknown at this time.

### Sequence variation that increases G4 formation potential increases RHPS4-mediated respiratory defects

**Figure 8:**
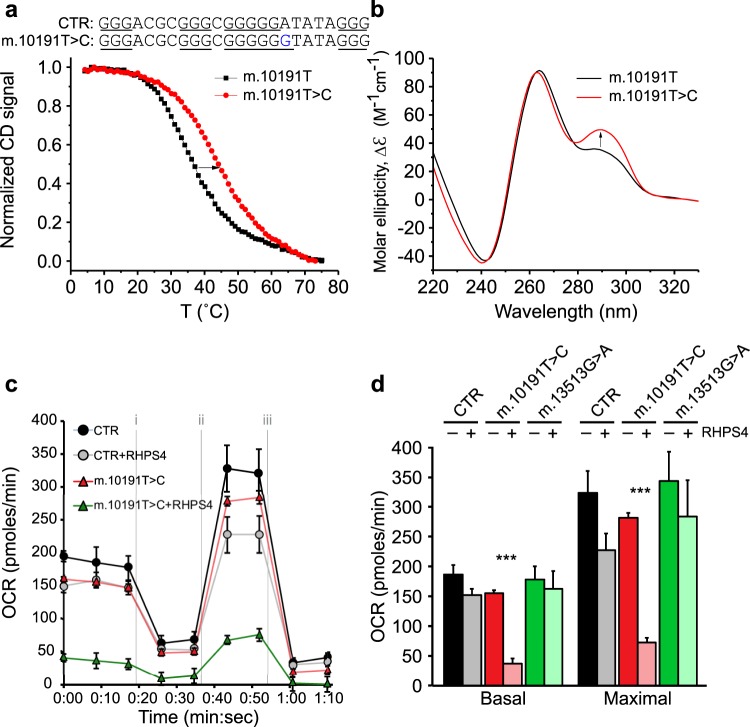
Cells harbouring a mtDNA variant that increases the G4 thermal stability and antiparallel characteristics show oxygen consumption defects upon RHPS4 exposure. (**a**) CD melting curves for control (CTR; black line) oligonucleotide sequence m.10184–10207 and m.10191T > C variant (red line) from heavy strand mtDNA. Arrow indicates the increase of melting temperature in the 10191T > C variant (**b**) CD spectra for CTR (black line) or the m.10191T > C variant (red line) show differences in parallel (peak at ~260 nm) and antiparallel (peak at ~295 nm) character. Arrow indicates the direction of change of the 10191T > C variant. All experiments were performed in 5 K^+^ buffer. (**c**) Example Seahorse Xf24 oxygen consumption rate (OCR) profile for control and m.10191T > C cell lines cultivated for 24 h in presence or absence of 1 µM RHPS4 (n = 5 well/line/RHPS4 condition). Treatments were as follows: (i) oligomycin; (ii) dinitrophenol; and (iii) rotenone. (**d**) Effects of RHPS4 exposure on basal and maximal oxygen consumption relative to control. Included are results from cells carrying the m.13513G > A variant, whose sequence change is not expected to alter G4 formation. No wells were excluded. All bar graph data are mean +/− SEM (p-values calculated against the untreated equivalent by one-way ANOVA with Sidak’s multiple comparison test: ***<0.001).

We tested whether mtDNA sequence could influence RHPS4-mediated effects on mitochondrial function (Fig. 8). To this end, we used the mtDNA variant m.10191T > C in the ND3 gene, which is associated with Leigh syndrome^44^. This sequence variant has one extra G in one of the G-rich stretches of the predicted G4-forming sequence of the heavy strand^45^ (Fig. 8a). The proximal region is present in the ND3 amplicon shown to be sensitive to RHPS4 in the PCR stop assay (Fig. 2e). We found that the presence of one extra G in m.10191T > C variant increased its thermal stability (Tm) from 34.8 to 43.0 °C (Fig. 8a). In addition, the mutation leads to an increase in the antiparallel character of the resulting G4-structure as indicated by the increased signal at 295 nm (Fig. 8b).

We next tested for RHPS4-mediated alterations to G4 structure and stability using two mtDNA G4 forming sequences, oligo A and B (Supplementary Fig. S9). We previously demonstrated that oligo A and B form predominantly parallel G4 structures as signified by the peak at 262 nm in circular dichroism (CD) spectra in the presence of 5 mM K^+^. K^+^ was chosen because it enhances G4 stability relative to Na^+^ or Li^+^ ^46^. The addition of 5 µM RHPS4 induced a marked antiparallel conformation in both oligo A and B sequences (Supplementary Fig. S9a,d), signified by the increase in peak intensity at 295 nm (and decrease of 264 nm peak). Oligo B was more sensitive to the presence of RHPS4. Consistent with our data, RHPS4 induced antiparallel G4 conformation in human telomeric DNA in the presence or absence of K^+^ ^21^. Thermodynamic stability of oligo A and B was tested in the presence of 2 eq of RHPS4 by CD. RHPS4 significantly increased melting temperatures of G4 structures formed by oligo A and B by 7.2 and 14.9 °C, respectively (Supplementary Fig. S9b,e). Our data are in agreement with the previous observation that RHPS4 stabilized human telomeric DNA and c-kit oncogene promoter sequences by ∼20 °C in similar experiments^21^.

To determine the strength of binding of RHPS4 to oligo A and to oligo B, we performed UV-Vis spectral titrations (Supplementary Fig. S9c,e). Curve fitting yielded binding constants (K_a_) of 0.5 × 10^6^/M, similar for both oligos with [G4]/[RHPS4] ratios of 1:3 for oligo A and 1:2 for oligo B. These rations suggest that up to three molecules of RHPS4 bind to oligo A and two molecules of RHPS4 bind to oligo B. We also observed significant red shift of 11.5 nm at 510 nm wavelength and enhancement of hyperchromicity by ∼22%, which demonstrate close, direct contact between G4 and RHPS4 (Supplementary Fig. S9c,f), consistent with end-stacking of RHPS4 onto the terminal G4-tetrad described previously^23^.

To test whether RHPS4 preferentially affects the m.10191T > C variant sequence in cells, we used cultured patient fibroblasts harbouring the m.10191T > C variant, the m.13513G > A variant (a non-G4 sequence) or normal control fibroblasts. We determined whether RHPS4 exposure differentially affected OXPHOS activity in these cells by measuring oxygen consumption under basal or maximal (uncoupled) conditions (Fig. 8c,d). RHPS4-untreated cells did not demonstrate significant differences in basal or maximal respiration among the different cell lines (Fig. 8c,d). However, low dose RHPS4 treatment (1 µM for 24 hrs) caused specific and profound decreases in oxygen consumption in the m.10191T > C variant fibroblasts with no effects observed in either control or m.13513G > A variant cell lines. In additional experiments, we selected m.10191T > C fibroblast cell lines whose mtDNA variant load (heteroplasmy) more closely matched a m. 13513G > A variant control line (~50%). We found that these m.10191T > C heteroplasmic lines also showed preferential OCR sensitivity when exposed to RHPS4 (Supplementary Fig. S10). Taken together, these data demonstrate the RHPS4-mediated effect on OCR in three distinct patient cell lines and highlight the potential susceptibility of specific mtDNA sequences to form G4 structures after RHPS4 treatment and to regulate mitochondrial function.

## Discussion

In this study, we used a well-established G4 ligand, RHPS4, to probe the roles of potential mitochondrial G-quadruplexes in mitochondrial gene expression. Based on the data presented here, we propose that RHPS4 translocates preferentially to the mitochondria most probably due to its positive charge, where it binds and is retained by mitochondrial G4 structures to interfere with transcription, replication, and as a result, impacts respiratory complex abundance. The differences between our observations of mitochondrial RHPS4 localization and the localization previously reported^24,25^ may be explained by microscopy artefacts described in the supporting data (Supplementary Figs S3 and S4); in addition, differences in cell lines used and exposure concentrations may also contribute. We reported that some nuclear localization occurred at higher RHPS4 concentrations, as we detected a mild activation of the DNA damage response at 10 µM. Gene expression responses consistent with nuclear DNA damage were absent at 2 µM RHPS4 exposure, dissociating nuclear DNA damage effects from the observed changes in mitochondria. Importantly, at 2 µM RHPS4 exposures, a decrease in the mtDNA abundance was observed, suggesting that nuclear DNA damage response was not a component of the mtDNA-related effects. This notion was further supported by mtDNA replication inhibition assays in a cell-free system with isolated mitochondria. All together, these findings suggest that low dose RHPS4 is a useful condition to study the effect of G4 stabilization on mitochondrial nucleic acids.

Although the specific sequences bound by RHPS4 in living cells are not known, clues arise from the data presented here. Our results demonstrate that RHPS4 preferentially interferes with polymerase amplification of specific regions of the mitochondrial genome, notably ND3. This region contains a predicted G4 that we demonstrated can fold *in vitro* into a G4 structure. A pathological variant in that ND3 G4 structure, m.10191T > C, increased G4 thermal stability and antiparallel character (Fig. 8a,b). We expect that a sequence with higher G4-forming potential, such as m.10191T > C, would form G4 more frequently *in vivo* thus would be more subject to RHPS4 binding. The antiparallel fold may also serve as a preferred substrate for RHPS4. Indeed, the m.10191T > C variant also enhances the cell sensitivity to RHPS4, exacerbating the mitochondrial respiratory defects, but the molecular alterations caused by RHPS4 remains the subject of speculation. It may be that RHPS4 stabilized the G4 structures that it binds to, but it is equally possible that the higher level of G4 formed by the variant sequence provides more opportunity for RHPS4 to have effects that extend beyond G4 stabilization, perturbing the G4 biology. Our combined results provide strong evidence that low concentrations of RHPS4 can be used to alter mitochondrial G4 structures.

Other mtDNA G4-forming sequences are likely to be involved in the RHPS4 effects on mitochondria. G4 structures can potentially form in single-stranded nucleic acids, which exist naturally during first strand replication of mtDNA. In contrast, the light strand that contains the potential i-Motif forming sequences is generally double-stranded throughout mtDNA replication. We predict that the compound binds to the displaced heavy strand, which occurs in the major arc region between the heavy and light strand origins (O_H_ and O_L_, respectively) containing two-thirds of the genome during first strand replication^15^. This same region acts as the template for HSP2-derived transcription, which is also affected by RHPS4. This region also contains the majority of mtDNA deletion breakpoints, which are associated with G4-forming sequences^6^, and targeted single-strand breaks in a G4-forming sequence downstream of O_L_ are sufficient to cause mtDNA deletions^11^. The spontaneous formation of G4 at that sequence may similarly arrest replication and increase deletion events. Further studies are required to reliably detect G4 structures *in situ* and identify sequences that form G4 with high-efficiency in normal and variant mtDNA sequences. Taken together, our current data suggest that conserved mtDNA strand asymmetry and consequent G4 sequence distribution can play a role in replication and gene expression.

The RNA priming of first strand mtDNA replication is thought to occur through mitochondrial RNA polymerase POLRMT-based LSP synthesis, where the nascent RNA can either elongate to generate full transcripts or terminate at the three conserved sequence blocks (CSB I, II, and III) that have been described *in vitro*^47^ to provide a 3′-OH for initiation of DNA replication. Recently, transcription termination at the human CSBII has been shown to depend on the formation of a DNA-RNA hybrid G4 structure^12,13^, which is opposed by the transcription factor TEFM^48,49^. As such, it might be expected that G4 stabilization would lead to transcription arrest at CSBII and LSP transcript depletion. Indeed, we observed a decrease in mature and unprocessed ND6 LSP transcripts at the low RHPS4 dose. Furthermore, the absence of RHPS4 effects at the mouse CSBII region may be due to the shorter central guanine run, consistent with the observation that the shorter central run causes RNA pausing less frequently than the longer variant^48^. Further investigations and the development of site-directed mtDNA mutagenesis technologies may be required to test the function of individual G4 sequences in the replication-transcription switch.

In our working model, formation of G4 structures may be a stochastic process, but their stability mandates dedicated activities for their resolution. Importantly, there are a number of G4-resolving helicases that can reduce formation of G4 structures. Among the most efficient G4 helicases is PIF1^50^. PIF1 G4-helicase activity is conserved from bacteria to mammals, and is essential for mtDNA stability in yeast^51^. PIF1 localizes both to the nucleus and mitochondria through alternative translation initiation mechanisms in both yeast and mammals^52,53^. Interestingly, skeletal muscle tissue isolated from PIF1 knockout mice shows precocious mtDNA deletions and decreased enzymatic activity in OXPHOS complex I^10^, whose subunits are encoded by 44% of the mitochondrial genome sequence. We do not yet understand the mechanisms underlying these phenotypes and the role of G4 structures in these processes.

Although the association of G4-forming sequences with mtDNA deletion breakpoints has been demonstrated^6,7,9^, the natural function of these structures in mitochondrial biology remains elusive^15^. Using a specific G4 ligand that stabilizes G4 structures in mitochondria, we have begun to understand the impact of these sequences on mitochondrial function. While our approach likely increases both the number and stability of G4 structures, other *in vivo* events could modulate G4 formation, thereby enabling G4 dynamics to act as a mitochondrial regulatory mechanism. Such modulators could involve ATP-dependent helicases (such as PIF1^10^), oxidative modification (8-oxoguanine and hydantoin^54,55^), or mtDNA variant (this study). This work will enable future studies to identify, quantify, and eventually target G4-forming sequences to increase our understanding of processes that regulate mtDNA transcription and replication as part of mitochondrial biogenesis and function.

## Materials and Methods

Chemicals, Cell culture and treatments, Cell viability measurement, Western blotting analysis, Interphase fluorescence *in situ* hybridization (FISH), RNA-Seq analysis, Live cell imaging, Fixed cell microscopy, Identification of mitochondrial sequences with G-quadruplex forming potential, and Oligonucleotides are located in Supplementary Information with appropriate references. All cell experiments were performed in accordance with appropriate approvals from the University of Pennsylvania, the University of Pittsburgh, or the University of Toronto.

### mtDNA measurements

Total genomic/mitochondrial DNA was isolated by sodium dodecyl sulfate lysis and proteinase K digestion from MEFs, HeLa, and C2C12 as previously described^56^. DNA was gently resuspended at 37 °C in Tris-EDTA (TE) buffer supplemented with RNAse A to remove the RNA contamination and DNA concentration was measured using an AccuBlue Broad range kit (Biotium, Fremont, CA). For measuring relative mouse mtDNA abundance, we designed TaqMan primer/probes for mitochondrial ND1 (VIC-labeled; primer:probe of 1:1) and nuclear TBP (FAM-labeled; primer:probe of 3:1), screened candidates for *in silico* primer compatibility, and validated the multiplex reaction by template serial dilution. The multiplex assessment of the relative abundance content of mtDNA was conducted by qPCR using TaqMan Fast Advanced Master Mix (ThermoFisher Scientific), 4.6 ng/reaction DNA and 5 μM of primer/probes in 10 μL final reaction volume and calculated by the ΔΔC_q_ method^57^. The qPCR amplification profile was: one cycle (95 °C for 20 sec) and 40 cycles (95 °C for 1 sec and 60 °C for 20 sec).

Measurement of major mitochondrial topoisomers was performed as previously reported^27,56,58,59^. In brief, total DNA was isolated as above, quantified by Nanodrop 1000 (ThermoFisher Scientific), and 2 µg of DNA resolved on 0.7% gel in 0.5X Tris/Borate/EDTA (TBE) buffer for 17 h at 40 V. DNA in gel was nicked, denatured, neutralized, and capillary transferred onto positively charged nylon (Hybond N+; GE Healthcare, Marlborough, MA). Membrane was rinsed, UV crosslinked, and air-dried. MtDNA isoforms were detected after random-hexamer probing and quantifying bands by phosphorimager analysis.

For the *in organello* replication assay, mitochondria were isolated from WT mouse liver by differential centrifugation^60^ and protein determined by the Qubit fluorescent assay (ThermoFisher Scientific). Mitochondria were pelleted and resuspended at 4 mg/ml protein concentration in incubation buffer (10 mM Tris-HCl pH 8.0, 20 mM sucrose, 20 mM glucose, 65 mM sorbitol, 100 mM KCl, 10 mM K_2_HPO_4_, 50 µM EDTA, 1 mM MgCl_2_, 5 mM glutamate, 5 mM malate, 1 mg/ml fatty acid free BSA, and 1 mM ADP)^30^. The mitochondrial preparation was divided into six 1 mL tubes, three for treatment with 2 µM RHPS4 and three without treatment. A dNTP mix containing 30 µL of each 10 mM dCTP, 10 mM dGTP, and 10 mM dTTP, as well as 12 µL α-^32^P-dCTP (3000 mmol/Ci), was then added at 17 µL per tube. Samples were gently rotated at 37 °C, collected at 30, 60, 90, and 120 min, spun down, and lysed in proteinase K/SDS buffer and total DNA isolated as described above. DNA was then resuspended in 50 µL of TE buffer with RNase A and dimethylurea antioxidant overnight, 25 µL was digested with Sac I restriction enzyme, and resolved on 0.6% agarose in 0.5X TBE for 2 h at 100 V/hr. DNA within the gel was nicked, denatured and transferred onto membrane, UV crosslinked, and exposed to phosphorimager for 24 h. Label incorporation into full-length mtDNA was determined using ImageQuant software package (GE Healthcare, Pittsburgh, PA). Total mtDNA was determined by random hexamer probing of full-length mtDNA.

### FRET melting assay

RHPS4 stabilization and selectivity for G4 DNA was determined using FRET assay with F21D as previously described^28^. In brief, F21D was annealed 24 h before the FRET study by heating the 0.25 μM F21D to 95 °C for 10 min in 5 K buffer (10 mM Li cacodylate pH 7.2, 5 mM KCl, and 95 mM LiCl) then slowly cooled to room temperature over three hours and stored at 4 °C overnight. RHPS4 was added in the concentration range from 0.4 to 4.0 µM bringing the final F21D concentration to 0.2 μM. Samples were thoroughly mixed and equilibrated at least for one hour before FRET measurements. Fluorescence measurements were taken every 1 °C from 15 °C to 95 °C, with temperature rate of 1 °C/min on a MJ research Chromo4 (now Bio-Rad, Hercules, CA). Melting temperature was determined from the first derivative of melting data. For FRET-based competition studies, 0.2 μM F21D sample incubated with 1.6 μM RHPS4 was supplemented with 4.8 to 96.0 μM calf thymus (CT) DNA as non-specific, double stranded DNA competitor. Negative control experiment included 0.2 μM F21D with 16 µM CT DNA in the absence of ligand. Melting temperature (Tm) was determined as a maximum of a peak of a first derivative of the melting data (associated error ± 0.5 °C). All samples were measured in technical duplicate per experiment and each experiment was repeated three times.

### Circular dichroism experiments

Circular dichroism (CD) wavelength scans were collected on oligos containing the 10191T and 10191T > C sequences to determine the extent and type of the folding in 5K buffer. The experiments were performed using AVIV 410 or 435 spectrometer equipped with a Peltier heating unit, with the following parameters: 2 nm bandwidth, 220–330 nm window, 1 sec average time, 10 °C temperature, and 3 scans. Resulting data were treated as described in our previous work^61^. CD melting experiments were performed by heating the samples from 4 to 90 °C and then back to test reversibility of the melting process. CD melting was performed using the following parameters: wavelength, 264 nm; bandwidth, 4 nm; T range: 4–95 °C; T step: 1 °C; dead band: 0.33 °C; equilibrating time, 0.5 min; averaging time, 20–30 sec. CD scans were collected after completion of the CD melt to assure that the samples had returned to equilibrium. Melting experiments were repeated at least three times. Data were analysed as described above for FRET melting.

### PCR stop assay

Polymerase stop assay was performed as reported^20^. Briefly, the experiment was conducted adding RHPS4 (0; 1; 2; 5; 10 µM) to a 25 µl reaction containing DreamTaq DNA Polymerase (ThermoFisher Scientific), 4.6 ng/reaction DNA, 0.2 mM dNTPs and 0.4 μM of each primer. The PCR was performed in a ProFlex thermal cycler (ThermoFisher Scientific) with the following PCR amplification profile: one cycle of 95 °C for 2 min; 30 cycles of 95 °C for 30 sec, 56 °C for 30 sec, and 72 °C for 30 sec. Products of amplification were run on a 2% agarose gel and detected with ethidium bromide fluorescence on a Bio-Rad ChemiDoc MP Imaging System (Bio-Rad, Carlsbad, CA). Quantitation was performed on three or more independent experiments.

### Measurement of mitochondrial PTP opening and mitochondrial membrane potential

The state of mitochondrial PTP was assessed using the quenching of calcein-AM fluorescence by cobalt chloride exposure^33^. Briefly, MEFs were treated with CsA 1 μM for 24 h, the media replaced and the cells pre-loaded with CoCl_2_ 1 mM for 30 min. Then cells were washed and incubated with 1 μM Calcein-AM (Thermo Fisher Scientific) for 30 min in the dark. MEFs were washed and resuspended in Hank’s Balanced Salt Solution (HBSS) prior analysis.

The mitochondrial membrane potential (ΔΨ_m_) was assessed using Tetramethylrhodamine methyl ester (TMRM) detection. Briefly, cells treated with CsA 1 μM for 24 h were washed and then incubated with TMRM (Thermo Fisher Scientific) 100 nM for 30 min. The fluorescence of 10,000 cells was measured by flow-cytometer (BD LSRFortessa Cell Analyzer, San Jose, CA). Data were analysed using FlowJo v7.6.5 software (Treestar, Ashland, OR).

### Transcript analysis

To quantify gene expression by real-time quantitative polymerase chain reaction (qRT-PCR), RNA was isolated from cell pellets with the RNeasy Mini Kit (Qiagen, Germantown, MD) and genomic DNA contamination was removed using DNA-free DNA Removal kit (ThermoFisher Scientific). Quality of the extracted RNA was assessed by 1% agarose gel electrophoresis and from the A_260nm_/A_280nm_ absorbance ratio (Nanodrop 1000, ThermoFisher Scientific). Next, cDNA was synthesized using the High Capacity cDNA Kit (ThermoFisher Scientific) with either random (provided by manufacturer) or specific primers (see Primers and probes section below). Finally, processed and unprocessed RNA expression levels were assessed using the ΔΔC_q_ method^57^. The primer/probe sets were designed, and their compatibility validated *in silico*. A serial dilution approach was used to validate the multiplex reaction. All qPCR reactions were carried out on either a StepOnePlus or QuantStudio 5 thermal cycler (ThermoFisher Scientific). All experiments were run in triplicate and the gene expression levels normalized to the B2M results.

### Measurement of oxygen consumption rate (OCR)

OCR of control and patient primary human fibroblasts treated with 0 or 1 μM RHPS4 for  24 h was measured using the XF-24 of XFe96 Seahorse system (Seahorse Bioscience, Billerica, MA). After  24 h incubation with vehicle (DMSO) or 1 µM RHPS4, cells were washed with pre-warmed assay medium and seeded at 5 × 10^3^ cells/well, five wells per group. For XFe96 experiments, 2.5 × 10^5^ cells per well were plated then incubated for 24 h with vehicle or 1 µM RHPS4. The basal respiration data were collected and the cells sequentially treated with 1 µM oligomycin, 150 µM dinitrophenol, and 160 nM rotenone as previously optimized for this system^62^. All experiments were performed in unbuffered DMEM supplemented with 5 mM glucose. In Fig. 8, the cell lines used were: normal human primary fibroblast; patient fibroblast carrying ~50% heteroplasmic variant load for m.13513G > A; patient fibroblast carrying ~90% variant load for m.10191 T > C. In Supplementary Fig. S10, the cell lines were from patients different from Fig. 8: m.13513G > A (~50% heteroplasmy), m.10191T > C (~50% heteroplasmy), and m.10191T > C (~55% heteroplasmy).

### UV-Vis binding studies

UV-Vis studies were performed to demonstrate direct ligand-DNA interaction between mtDNA oligo A or oligo B and RHPS4 using a Cary 300 Varian spectrophotometer with a Peltier-thermostated cuvette holder (error of ± 0.3 °C) in 5 K buffer. The advantage of this experiment over FRET assay is the absence of fluorescence tag on DNA. Titrations were performed by stepwise additions of annealed oligo A or oligo B to a 0.3 mL solution of 20 µM RHPS4 in a 1 cm quartz cuvette. Resulting solution was mixed and equilibrated for two min. UV-Vis spectra were acquired in 350–670 nm range. Titration was deemed complete when the spectra collected after three successive additions of G4 oligonucleotides were nearly superimposable. At this point, typical [G4]/[RHPS4] was ~ 3. Spectra were corrected for dilution effects and the resulting data were treated as described in detail in our earlier work^61^. Direct fitting of UV-Vis data (assuming two-state equilibrium) was used to obtain the values of binding constant, K_a_. Hypochromicity (% H, decrease in signal intensity) and red shift (change in peak position) were extracted from UV-Vis data as described in^61^. Titrations were repeated at least three times.

### Primers and probes

For the mature RNA assay, standard qPCR thermal parameters were used: one cycle of 95 °C for 20 sec then 40 cycles of 95 °C for 1 sec and 60 °C for 20 sec. For the parental RNA assays, the optimized conditions were as follows: L-ND1 or F-RNR1-V-RNR2 mouse gene expression, one cycle of 95 °C for 10 min then 40 cycles of 95 °C for 20 sec, 56 °C for 20 sec and 60 °C for 20 sec; ND6-E-CYTB or E-ND6 mouse gene expression, one cycle of 95 °C for 10 min then 40 cycles of 95 °C for 20 sec, 60 °C for 20 sec and 60 °C for 20 sec.

### Statistical analysis

Statistical analysis was performed by one-way ANOVA with Dunnett’s posthoc analysis or Student two-tailed test using GraphPad Prism software (GraphPad, La Jolla, CA). P-values of < 0.05 were accepted as a significant difference. All data are expressed as mean +/− standard error of the mean (SEM).

### Animals

Balb/cJ mice were purchased from Jackson Laboratories with institutional animal care and use committee (IACUC) approval from the University of Pennsylvania or the University of Pittsburgh. All methods were performed in accordance with Guide for the Care and Use of Laboratory Animals.

## Supplementary information

Supplementary Information

Supplementary Dataset and Figures

Supplementary Dataset S1

Supplementary Dataset S2

Supplementary Dataset S4

Supplementary Dataset S5

Supplementary Figure S3

Supplementary Figures S5

## Data Availability

RNA-Seq data are available from GEO using the token GSE103116.
