# Supplementary material for: G-quadruplex dynamics contribute to regulation of mitochondrial gene expression
Source: Sci Rep. 2019 Apr 3;9:5605. doi: 10.1038/s41598-019-41464-y (PMC6447596; doi:10.1038/s41598-019-41464-y)
Supplement: Supplementary file 1 — Supplementary Information [file 41598_2019_41464_MOESM1_ESM.docx]

TITLE: **G-quadruplex dynamics contribute to regulation of mitochondrial gene expression.**

M. Falabella^1,†^, J. E. Kolesar^2,†^, C. Wallace^3^, D. de Jesus^1^, L. Sun^1^, Y. V. Taguchi^2^, C. Wang^2^, T. Wang^4^, I. M. Xiang^5^, J. K. Alder^6^, R. Maheshan^7^, W. Horne^8^, J. Turek-Herman^5^, P. J. Pagano^1^, C. M. St. Croix^3^, N. Sondheimer^7^, L. A. Yatsunyk^5^, F. B. Johnson^9^, B. A. Kaufman^1,*^

**Supplementary Information**

**Chemicals**

Cell culture reagents: Dulbecco’s Modified Eagle’s Medium (DMEM; HyClone, Logan, UT); trypsin-EDTA 1X 0.25% (Corning, Tewksbury, MA); bovine serum albumin (BSA) and ethidium bromide (EtBr; Sigma-Aldrich, St. Louis, MO); fetal bovine serum (FBS); fetal calf serum (FCS); phosphate buffered saline (PBS) and penicillin/streptomycin (ThermoFisher Scientific, Waltham, MA). Chemicals: 3,11-Difluoro-6,8,13-trimethylquino[4,3,2-*kl*]acridinium methylsulfate (RHPS4; kind gift of Dr. Marc Hummersone, Pharminox Ltd., Nottingham, UK and purchased from Tocris, Bristol, UK); BRACO-19 (Organic Synthesis Facility at Fox Chase, Philadelphia, PA); N-methyl mesoporphyrin IX (NMM; Frontier Scientific, Logan, UT); Phen-DC3 (kindly provided by Dr. David Monchaud and Dr. Marie-Paule Teulade-Fichou, Curie Institute, Paris, France); FCCP (trifluoromethoxy carbonylcyanide phenlhydrazone; Sigma-Aldrich); and cyclosporin A (CsA; Fisher Scientific, Pittsburgh PA). All primers and probes are from Integrated DNA Technologies (Coralville, IA). All other chemicals were reagent grade or better.

**Cell culture and treatments**

Mouse embryonic fibroblasts (MEFs), C2C12 myoblasts, HeLa, 143B ρ^0^ and 143B ρ^+^ cells were grown in DMEM medium supplemented with 25 mM glucose, 4 mM glutamine, 110 mg/ml pyruvate, 10% (v/v) FBS:FCS (1:1), 100 U/ml penicillin and 100 mg/ml streptomycin at 37°C under standard conditions (5% CO_2_; ambient O_2_; 95% relative humidity). 143B ρ^0^ and 143B ρ^+^ medium was further supplemented with 0.2 mM uridine. HeLa cells were verified by single nucleotide polymorphism genotyping at the University of Pittsburgh HSCRF Genomics Research Core. All cell lines were mycoplasma free. Sub-confluent wild type MEFs and HeLa cells were cultivated in media containing RHPS4 at indicated concentration (0, 1, 2, or 10 µM) and time (16 or 24 h). Drug wash-out was performed by pre-warmed media replacement. EtBr treatment was conducted on sub-confluent MEFs for 24 h in the presence of 25 or 250 ng/ml compound. Cells were collected by trypsinization, pelleted at 200 x g for 5 min, washed with PBS, and flash frozen in liquid nitrogen for later DNA or RNA preparation.

**Cell viability measurement**

Cell viability was assessed by crystal violet assay as reported in ^1^. Briefly, MEFs cells were seeded in 96 well plate at 5 x 10^3^ cells/well, eight wells per group. After 24 h incubation with RHPS4 (0; 2; 10 µM) or CsA (0; 0.5; 1; 2; 10 µM), the medium was discarded, the cells washed with PBS and incubated with a crystal violet staining solution (0.5%) at room temperature for 20 min. The cells were then washed twice with water and dried for 2 h. Dye was released by 20 min incubation in 200 µl methanol and the optical density of stained cells was read at 570 nm using a BioTek Sinergy 4 Hybrid Multi-Mode Microplate Reader (BioTek, Winooski, VT; USA).

**Western blotting analysis**

Cell pellets were lysed in RIPA buffer (50 mM Tris-HCl, pH 7.4, 150 mM NaCl, 0.25% sodium deoxycholate, 1 mM EDTA, 1% NP-40, 1X Complete protease inhibitor cocktail [Roche Molecular Diagnostics, Pleasanton, CA], and phosphatase inhibitor cocktail [ThermoFisher Scientific]). Cell extracts were separated on a 15% (γ-H2AX) or 4-12%(OXPHOS subunits) Bis-Tris polyacrylamide gel and proteins analyzed by immunoblotting using primary antibodies against the following proteins: Ser140-phospho-H2AX (γ-H2AX;ThermoFisher Scientific); ATP5A, UQCRC2, MTCO1, SHDB and NDUFB8 (Total OXPHOS Rodent WB Antibody Cocktail; Abcam); SDHA (Abcam); TFAM (PhosphoSolutions, Aurora, CO); β-actin (Santa Cruz Biotechnology, Santa Cruz, CA) and GAPDH (EMD-Millipore, Billerica, MA) followed by IR dye labelled secondary antibodies (Li-Cor, Lincoln, NE). Signal intensity was detected with the Li-Cor Odyssey infrared imager at 680 and 800 nm and processed with manufacturer’s software.

**Interphase fluorescence in situ hybridization (FISH)**

Interphase Fluorescence *in situ* Hybridization (FISH) was carried out as described in ^2^ with the following modifications: the PNA telomere probe was conjugated to AlexaFluor 647 and the images taken on Nikon A1 Spectral Confocal Laser Microscope System and processed in Nikon NIS Elements software (Nikon Inc., Melville, NY).

**RNA-Seq analysis**

For RNA-Seq experiments, total RNA concentration and quality from each sample was assessed using Qubit 2.0 fluorometer (ThermoFisher Scientific) and Agilent TapeStation 2200 (Agilent, Santa Clara, CA). Total RNA libraries were generated using Illumina TruSeq Stranded Total RNA Sample Preparation Guide Rev. E (Illumina, San Diego, CA). In brief, this process depletes nuclear ribosomal RNA (rRNA) and then fragments the remaining RNA, which was converted into first strand cDNA using reverse transcriptase and random primers. The second strand cDNA was generated using DNA polymerase I and RNase H. The cDNA fragments were further processed for adapter ligation, and then enriched with PCR to create the final cDNA library.

The cDNA libraries were validated using Illumina-compatible DNA primers (KAPA Biosystems, Wilmington, MA) and Qubit 2.0 fluorometer. Quality was examined using Agilent Tapestation 2200. The cDNA libraries were pooled at a final concentration 1.8 pM. Cluster generation and 75 bp paired read dual-indexed sequencing was performed on Illumina NextSeq 500.

Quality control for raw fastq files were performed with FastQC ^3^, the low quality reads and 3’ adapters were trimmed with Trim Galore! and Cutadapt ^4,5^. The trimmed reads were aligned to reference genome (mm10) with the RNA-seq aligner STAR ^6^, and the resultant bam files were converted to bigWig format using the *bam2wig* function in RSeQC ^7^ for the visualization of read coverage in genome browser, such as IGV ^8^. Subsequently, gene expression in each sample were quantified as the number of read fragments that were uniquely mapped to genes using featureCounts ^9^. The raw count was then normalized as FPM (Fragments Per Million mapped) to eliminate the influence of sequencing depth. Gene differential expression analysis was performed on the raw count table using DESeq2 ^10^. Genes with FDR < 0.01 and fold-change > 1.5 were determined as differentially expressed genes (DEGs). Statistical enrichment of the DEGs in the KEGG pathways was performed with Fisher’s exact test.

**Identification of mitochondrial sequences with G-quadruplex forming potential**

Reference mouse mtDNA sequence (NC_005089) was subjected to G4 Hunter QFP prediction as using 25 nt window and a threshold of 1.0 ^11^. Merged outputs are shown with any overlapping results adjusted to be discrete.

**Live cell imaging**

To detect the RHPS4 endogenous fluorescence, HeLa and MEF cells were seeded on 35 mm glass bottom dishes (MatTek Corporation, Ashland, MA) and incubated with 1 µM RHPS4 overnight prior to imaging. The dish was inserted in a closed, thermo-controlled (37ºC) stage top incubator (Tokai Hit Co., Shizuoka-ken, Japan) atop the motorized stage of an inverted Nikon TiE fluorescent microscope equipped with a 60X oil immersion optic (Nikon, CFI PlanFluor, NA 1.43) and NIS Elements Software. RHPS4 was excited using either the 470 nm or the 555 nm line of a Lumencor diode-pumped light engine (SpectraX, Lumencor Inc., Beaverton, OR) and detected using ET-GFP and ET-TRITC emission filter (Chroma Technology Corp., Bellows Falls, VT) and ORCA-Flash4.0 sCMOS camera (HAMAMATSU Corporation, Bridgewater, NJ).

For the RHPS4 mitochondrial localization, HeLa or MEFs cells were treated in culture with 1 µM RHPS4 overnight, washed with PBS, and incubated with MitoTracker Deep Red FM (ThermoFisher Scientific) probe in the dark for 15 min. The RHPS4 signal was detected using 561 nm excitation and TRITC emission filters. Images were captured on a Nikon Ti equipped with a 60X (1.4 NA) optic using a Sweptfield confocal scanhead (35 micron slit; Prairie Instruments, Middleton, WI), a Photometrics Evolve 512 camera (Tucson, AZ) and NIS Elements software. Binary masks were generated in NIS Elements and binary math used to calculate the volume of protein within the mitochondria compartment, as defined by the MitoTracker Deep Red signal. For the 3D rendering of RHPS4 co-localization with mitochondria, live cells were imaged using a 60X (1.40 NA) objective on a Nikon Ti inverted microscope equipped with a Tokai-Hit environmental chamber and Hamamatsu Flash 4.0 CMOS camera and processed in Nikon NIS Elements software.

To assess co-localization of RHPS4 with mitochondria after uncoupling, 143B ρ^+^ and ρ^0^ cells were seeded on 35 mm glass bottom dishes and incubated overnight with 1 µM RHPS4 prior to imaging. The cells were mounted in a Tokai-Hit environmental chamber atop the stage of a Nikon Ti inverted microscope equipped with a Sweptfield confocal head and imaged using a 60x 1.40 N.A. objective, and Photometrics Evolve EMCCD camera. Cells were imaged at 30 sec intervals for 15 min to establish a baseline level of fluorescent signal and observe the amount of photobleaching due to imaging conditions. Immediately after baseline levels were established, 1 µM trifluoromethoxy carbonylcyanide phenlhydrazone (FCCP) compound was added to the culture media and the cells were observed for 15 min at 30 sec intervals with the same imaging conditions as in the baseline experiment. Data were processed using NIS Elements software.

**Fixed cell microscopy**

MEF cells were grown on microscope coverglass (Fisher Scientific) overnight and incubated with 30 μM RHPS4 (Tocris Bioscience) for 30 min prior to permeabilization with ice-cold methanol for 5 min. For the immunovisualization of γ-H2AX, MEFs were treated with 0, 2 and 10 μM RHPS4 overnight, gently washed twice and fixed with 2% paraformaldehyde in PBS for 15 min at room temperature. Cells were then permeabilized with 0.25% Triton X-100, and blocked with 20% serum and 1% BSA in PBS (PBB) for 45 min at room temperature. Next, cells were incubated with the specific primary antibody for Ser140-phospho-H2AX (ThermoFisher Scientific) for 1 h at room temperature. Cells were then washed three times with PBB followed by incubation with the secondary antibody Alexa Fluor 647 (Invitrogen) for 1 h at room temperature. After washing, the nuclei were stained with Hoechst (Bisbenzimide; Sigma-Aldrich Co.) Images were taken using a Nikon A1 Spectral Confocal Laser Microscope System and processed in Nikon NIS Elements software (Nikon).

**Oligonucleotides**

The following primers were used for in vitro G-quadruplex formation assays:

F21D **(**5’-6-FAM-G_3_(TTAG_3_)_3_-Dabcyl-3’);

Oligo A (5’-GGATGGGGTGGGGAGG-3’);

Oligo B (5’-GGGGGATGCGGGGG-3’)

Primers used to perform the PCR stop assay were:

mt ND3 primer 1 (5’-AAAATCCACCCCTTACGAGT-3’)

mt ND3 primer 2 (5’-TATTGGCTAAGAGGGAGTGG-3’)

mt COX1 primer 1 (5’-GGTTCGATTCCTTCCTTTTT-3’)

mt COX1 primer 2 (5’-GCCTGACTGGCATTGTATTA-3’)

mt non G4 primer 1 (5’-GCACTCGTAAGGGGTGGAT-3’)

mt non G4 primer 2 (5’-TCGAGTCTCCCTTCACCATT-3’)

The specific primers used for the cDNA synthesis were:

E-ND6/ND6 (5’-TCCAAACACAACCAACATCC-3’);

F-RNR1-V-RNR2 (5’-GGTGTAGGCCAGATGCTTTAAT-3’);

ND1-IQM (5’-AAGAGGGCTTGAACCTCTATAA-3’);

ND6-E-Cytb (5’-GGCAGGTAGGTCAATGAATGAGTG-3’);

B2M (5’-CCGTTCTTCAGCATTTGGATTT-3’).

### For the mature RNA assay, the mouse probes ND6 (Mm04225325_g1), RNR1 (Mm04260177_s1), RNR2 (Mm04260181_s1), ND1(Mm04225274_s1), ND2 (Mm04225288_s1), COX1(Mm04225243_g1), COX2 (Mm04225288_s1), ATP6 (Mm03649417_g1), COX3 (Mm04225261_g1), ND3 (Mm04225292_g1), ND4L (Mm04225306_g1), ND4 (Mm04225294_s1), ND5 (Mm05688717_g1), CYTB (Mm04225271_g1) were from ThermoFisher Scientific.

### The primers and probes used for the parental RNA assays were purchased from Integrated DNA Technologies (IDT):

E-ND6 probe (5’-/56-FAM/TTGGTTGGT/ZEN/TGTCTTGGGTTAGCA-3’)

E-ND6 primer 1 (5’-GTCATTGGTCGCAGTTGAATG-3’)

E-ND6 primer (5’-ACCTCCATAAATAGGTGAAGGC-3’)

F-RNR1-V-RNR2 probe (5’-/56-FAM/AAACACAAA/ZEN/GGTTTGGTCCTGGCC/-3’)

F-RNR1-V-RNR2 primer 1 (5’-GCTTAATAACAAAGCAAAGCACTG-3’)

F-RNR1-V-RNR primer 2 (5’- TCTATGGAGGTTTGCATGTGTA-3’)

L-ND1 probe (5’-/56-FAM/AGGATTTGA/ZEN/ACCTCTGGGAACAAGGT/-3’)

L-ND1 primer 1 (5’-AGCCAGGAAATTGCGTAAGA-3’)

L-ND1 primer 2 (5’-GGGACGAGGAGTGTTAGGATA-3’)

ND6-E-CYTB probe (5’-/56-FAM/TTGGTTGGT/ZEN/TGTCTTGGGTTAGCA/3’)

ND6-E-CYTB primer 1 (5’-AAACAACCAACAAACCCACTAAC-3’)

ND6-E-CYTB primer 2 (5’-GCAGTTGAATGCTGTGTAGAAATA-3’)

B2M probe (5’-/5HEX/TTCAAGTAT/ZEN/ACTCACGCCACCCACC-3’)

B2M primer 1 (5’-ACGTAGCAGTTCAGTATGTTCG-3’)

B2M primer 2 (5’-GGTCTTTCTGGTGCTTGTCT-3’)

**References**

1. Feoktistova, M., Geserick, P. & Leverkus, M. Crystal violet assay for determining viability of cultured cells. *Cold Spring Harb. Protoc.* **2016,** 343–346 (2016).

2. Cesare, A. J., Heaphy, C. M. & O’Sullivan, R. J. Visualization of telomere integrity and function in vitro and in vivo using immunofluorescence techniques. *Curr. Protoc. Cytom.* **2015,** 12.40.1-12.40.31 (2015).

3. Andrews, S. & others. FastQC: a quality control tool for high throughput sequence data. (2010).

4. Krueger, F. Trim Galore!: A wrapper tool around Cutadapt and FastQC to consistently apply quality and adapter trimming to FastQ files. (2015).

5. Martin, M. Cutadapt removes adapter sequences from high-throughput sequencing reads. *EMBnet.journal* **17,** 10 (2011).

6. Dobin, A. *et al.* STAR: Ultrafast universal RNA-seq aligner. *Bioinformatics* **29,** 15–21 (2013).

7. Wang, L. *et al.* RSeQC: quality control of RNA-seq experiments. *Bioinformatics* **28,** 2184–2185 (2012).

8. Thorvaldsdóttir, H., Robinson, J. T. & Mesirov, J. P. Integrative Genomics Viewer (IGV): High-performance genomics data visualization and exploration. *Brief. Bioinform.* **14,** 178–192 (2013).

9. Liao, Y., Smyth, G. K. & Shi, W. FeatureCounts: An efficient general purpose program for assigning sequence reads to genomic features. *Bioinformatics* **30,** 923–930 (2014).

10. Love, M. I., Huber, W. & Anders, S. Moderated estimation of fold change and dispersion for RNA-seq data with DESeq2. *Genome Biol.* **15,** 550 (2014).

11. Bedrat, A., Lacroix, L. & Mergny, J. L. Re-evaluation of G-quadruplex propensity with G4Hunter. *Nucleic Acids Res.* **44,** 1746–1759 (2016).
