# Supplementary material for: G-quadruplex dynamics contribute to regulation of mitochondrial gene expression
Source: Sci Rep. 2019 Apr 3;9:5605. doi: 10.1038/s41598-019-41464-y (PMC6447596; doi:10.1038/s41598-019-41464-y)
Supplement: Supplementary file 2 — Supplementary Dataset and Figures [file 41598_2019_41464_MOESM2_ESM.pdf]

TITLE: **G-quadruplex dynamics contribute to regulation of mitochondrial gene expression.**

M. Falabella[†], J. E. Kolesar[†], C. Wallace, D. de Jesus, L. Sun, Y. V. Taguchi, C. Wang, T. Wang, I. M. Xiang, J. K. Alder, R. Maheshan, W. Horne, J. Turek-Herman, P. J. Pagano, C. M. St. Croix, N. Sondheimer, L. A. Yatsunyk, F. B. Johnson, B. A. Kaufman[*]

[†]These authors contributed equally to this manuscript.

[*]To whom correspondence should be addressed. E-mail: bkauf@pitt.edu. 200 Lothrop St. BST E1241, Pittsburgh, PA 15261.

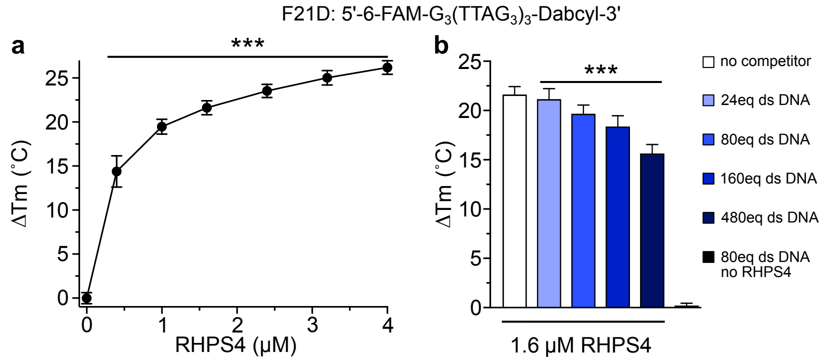

Supplementary Figure S1. RHPS4 is a G-quadruplex ligand that stabilizes antiparallel structures in G4-forming mtDNA sequences. (a) FRET-melting profile of 0.2 μM F21D in the presence of increasing concentration of RHPS4 from 0 to 4 μM. F21D is a fluorescently labelled G4-forming human telomeric DNA. (b) FRET competition assay showing the inability of dsDNA to efficiently compete with F21D for RHPS4 binding. Even in the presence of 480 eq of dsDNA RHPS4 stabilizes F21D by 15.3 +/- 0.9 ˚C. Negative control consists of 0.2 μM F21D, no ligand, and 80 eq of dsDNA. P-values for Panel (a) and (b) were calculated by one-way ANOVA with Dunnett's posthoc analysis: ***<0.001).

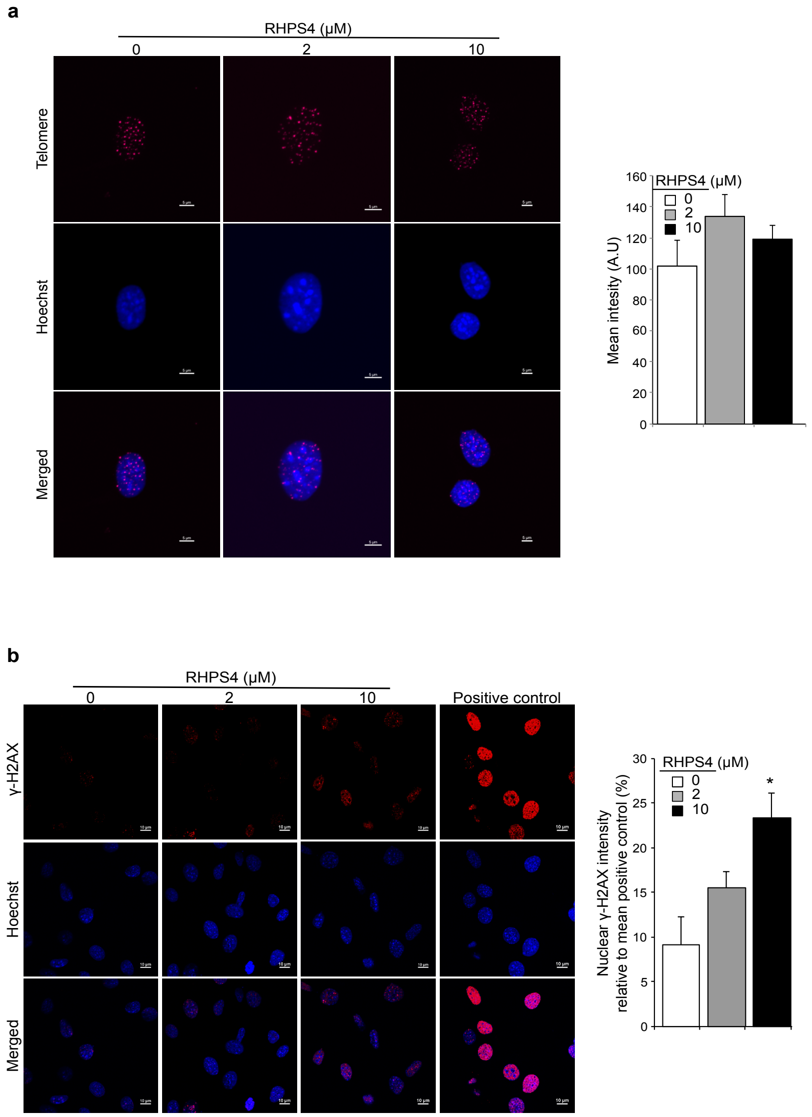

Supplementary Figure S2. Low doses of RHPS4 do not induce nuclear DNA damage. (a) Immuno-FISH representative confocal images, performed with an anti-telomeric PNA probe, of MEFs treated with 2 and 10 μM RHPS4 for 24 h. Three fields per conditions were analyzed. Data are mean +/- SEM. (b) Immunofluorescent confocal microscopy on fixed MEFs incubated with 2 and 10 μM RHPS4 for 24 h and stained for γ-H2AX. Positive control cells were treated with 100 μM H_2O_2 for 30 min. Mean signal intensity was normalized to % positive control. Experiment was run in triplicate and three fields of images were collected and processed per condition. Shown are mean values +/- SEM (p-values calculated by one-way ANOVA: *<0.05)..

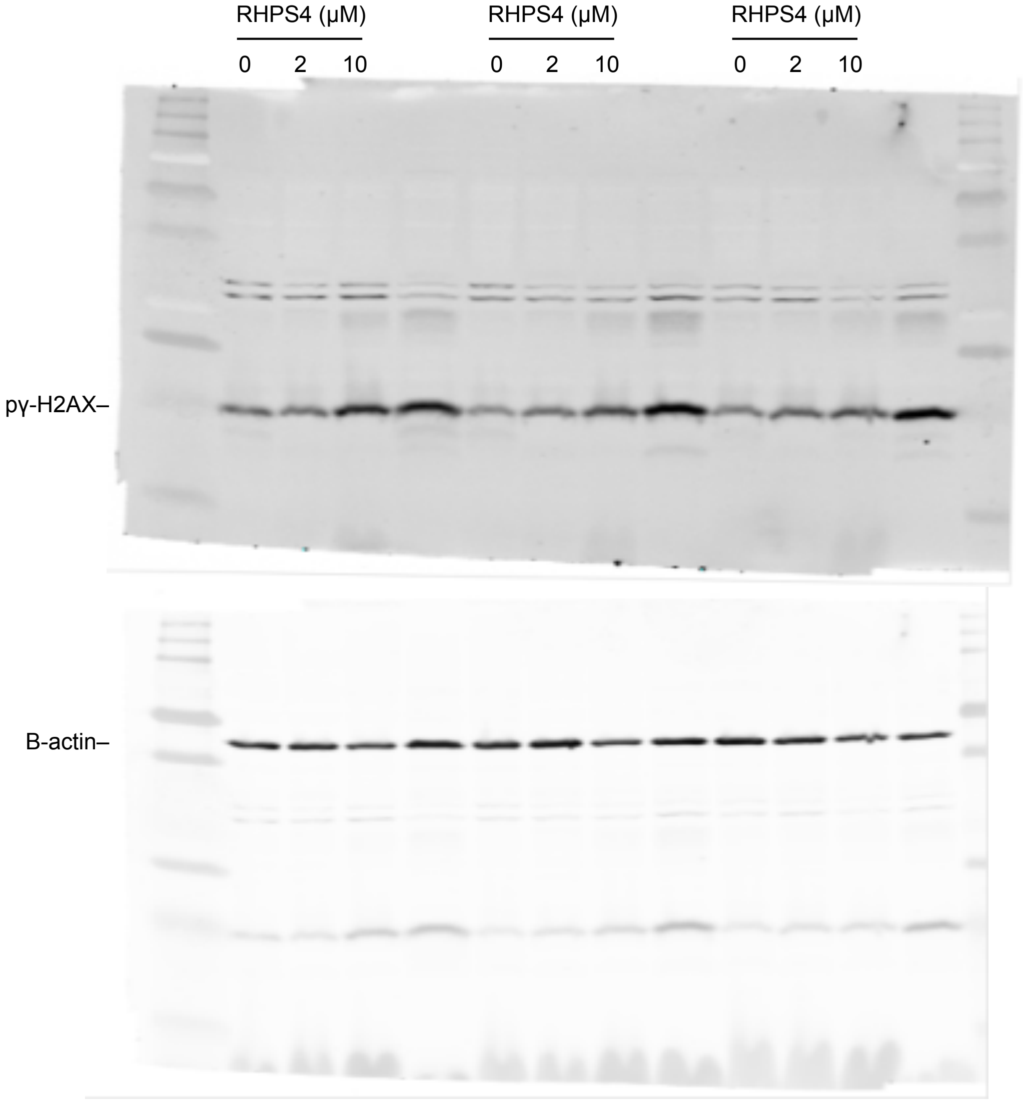

Supplementary Dataset S3. Raw data from phospho-γ-H2AX western.

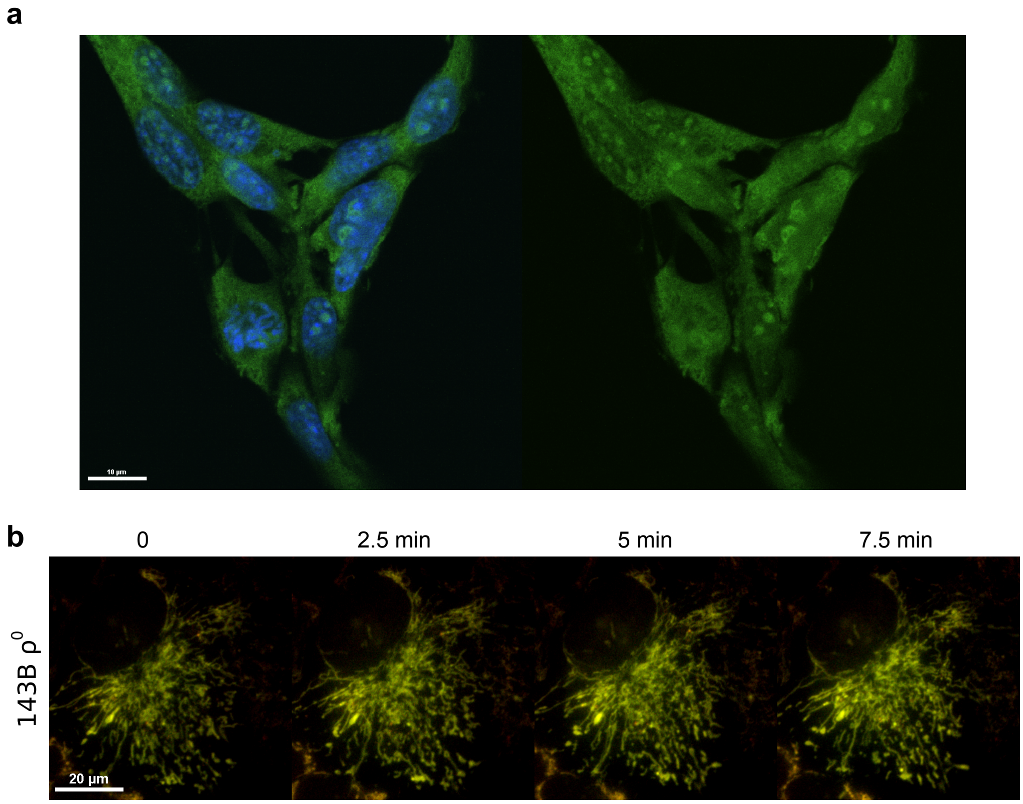

Supplementary Figure S4. Photobleaching and alcohol fixation controls. (a) Confocal microscopy image of MEF cells treated with 30 µM RHPS4 (green) for 30 min and permeabilized with cold methanol for 5 min. Left image shows nuclear DAPI staining (blue) with RHPS4, right image shows only RHPS4. (b) Live cell confocal images of 143B ρ0 cells incubated with 1 µM RHPS4 overnight. The time course of images show RHPS4 the overlay (yellow) of endogenous fluorescence from FITC channel (green) and TRITC channel (red) prior to treatment with FCCP in Figure 3c. Although the fluorescence is photostable, the nucleolar signal increases with repeated light exposure

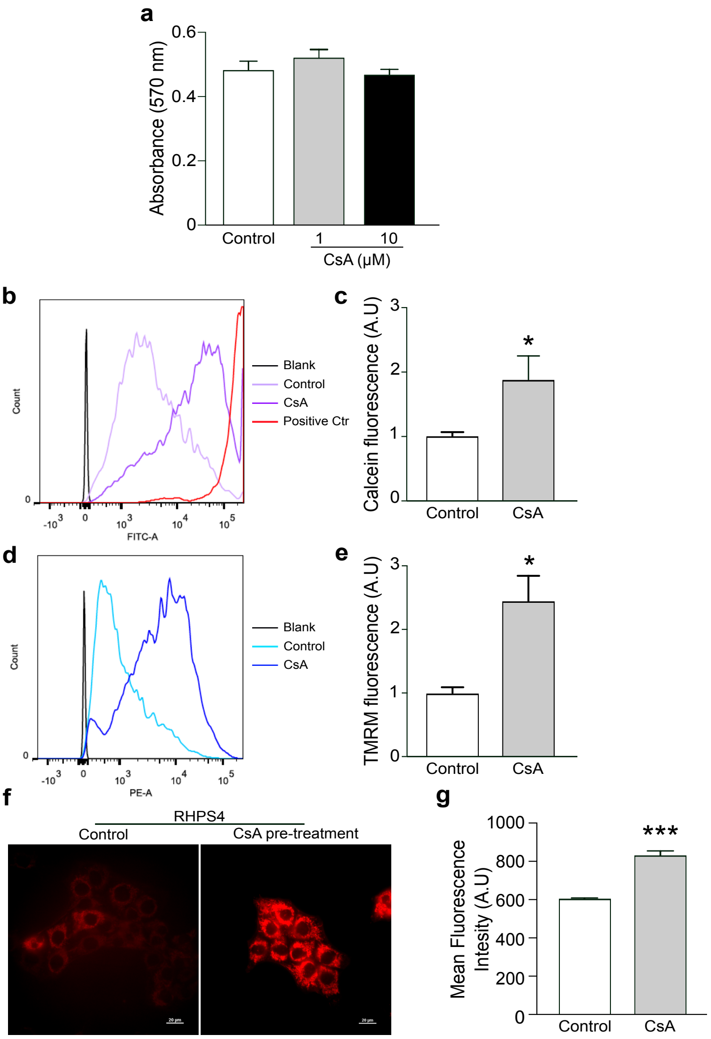

Supplementary Figure S6. Pre-treatment with the mitochondrial permeability transition pore inhibitor cyclosporine A (CsA) does not prevent RHPS4 uptake. (a) Bar graph of crystal violet viability assay on MEFs incubated with different CsA concentrations for 24 h. Shown are mean values +/- SEM (n=3-5; p-values calculated by one-way ANOVA: *<0.05, **<0.01, ***<0.001). (b) Representative flow cytometer histogram of the intensity of calcein signal after CoCl2 stimulation in the absence (lilac line) or presence (violet line) of 1 µM CsA. Blank and positive control are represented by black and red lines, respectively. (c) Quantification of calcein fluorescence in control and CsA-treated cells from panel b. (d) Representative flow cytometer histogram of the TMRM signal intensity detected in the absence (cyan line) or presence (blue line) of 1 µM CsA and blank (black line). (e) Quantification of TMRM fluorescence in control and CsA-treated cells from panel d. Bar graph data shown as mean normalized to untreated sample values +/- SEM (n=3-6; p-values calculated by unpaired t-test: *<0.05) (f) Live cell confocal images of RHPS4 endogenous fluorescence in the TRITC channel (red) in the control (left) or after CsA pre-treatment (right). MEF cells were pre-treated with 1 µM CsA for 24 h, washed and incubated with RHPS4 1 µM for 8 h. (g) Quantification of the RHPS4 endogenous fluorescence. Five fields of images were collected and processed per condition. Data are mean +/- SEM.

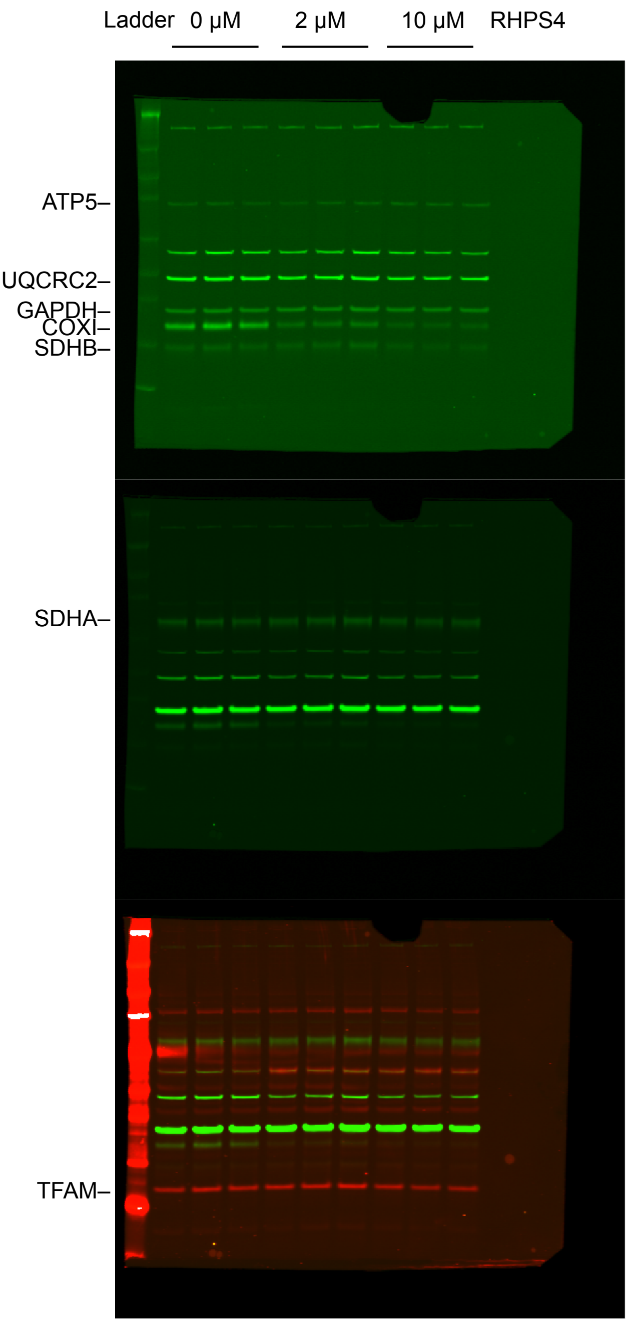

Supplementary Dataset S4: Raw Licor files images for quantitation from Figure 4

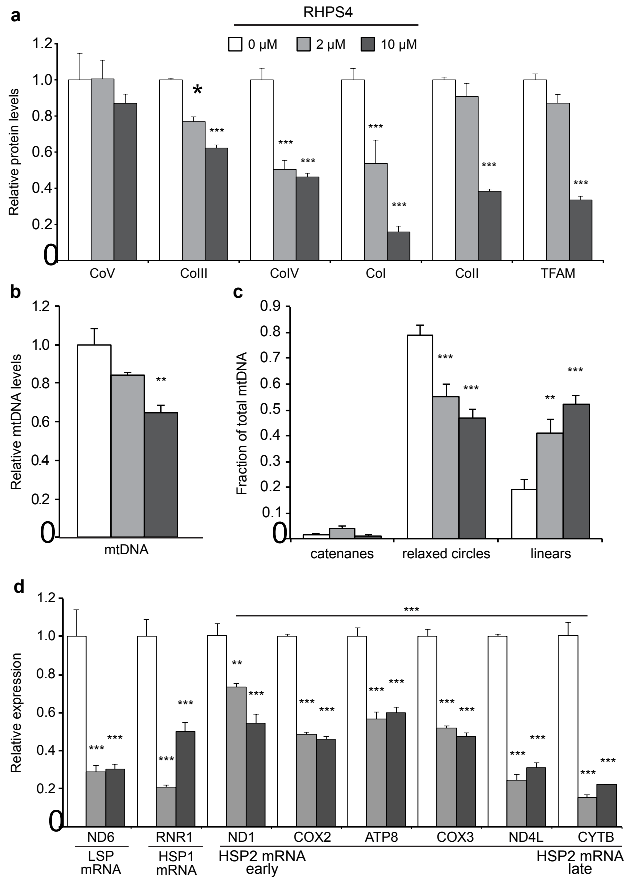

Supplementary Figure. S7. RHPS4 causes differential mitochondrial protein depletion, transcript deficiency, and decreased circular mtDNA in fully differentiated myotubes. C2C12 myotubes were treated for 24 h with 0, 2, and 10 μM RHPS4. (a) At low dose RHPS4 (2 μM), NDUFB8 (Complex I) and mt-CoI (Complex IV) are preferentially depleted. More extensive depletion of all proteins is detected in cells exposed to 10 μM RHPS4. (b) Myotubes show a significant mtDNA depletion after a 24 h exposure to 10 μM RHPS4. (c) As observed in MEFs, 1D-IMAGE analysis of myotubes show increased linear mtDNA content. (d) Representative mitochondrial transcripts are significantly affected by RHPS4 exposure. As observed with MEFs, transcripts distal to HSP2 (HSP2 mRNA late) are preferentially affected by RHPS4 exposure compared to those closer to the HSP2 (HSP2 mRNA early). Data are mean normalized to untreated sample values +/- SEM (n=3-6; p-values calculated by one-way ANOVA: *<0.05, **<0.01, ***<0.001).

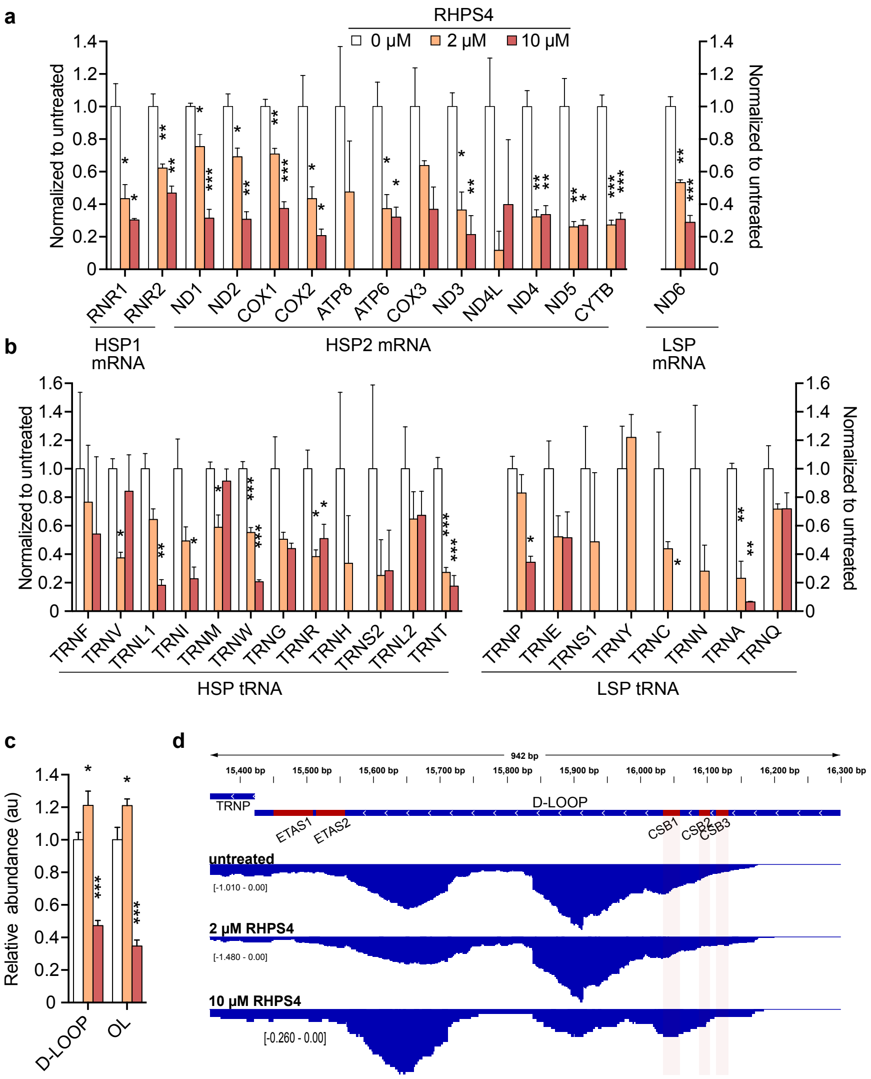

Supplementary Figure S8. Strand-specific RNA-SEQ analysis in MEFs confirms that RHPS4 treatment induces mitochondrial transcription defects prior to significant mtDNA depletion. (a) Strand-specific RNA-SEQ after depletion of cytoplasmic ribosomes showed that RHPS4 exposure decreases transcript levels more extensively with distance from the promoter in agreement with qRT-PCR data. Below the genes are the indicated promoters as in Fig. 5. (b) HSP tRNA levels show a similar trend of depletion. Low sequence count of tRNAs were noted. (c) Strand-specific D-loop RNA is not depleted by RHPS4 prior to genome depletion. Slight increases in D-loop mapped RNA reads are consistent with the maintenance of mtDNA abundance at 2 μM RHPS4 exposure. (d) The region around the CSB2 G4-forming sequence does not show alterations at low RHPS4 exposure. Top diagram shows mtDNA sequence map for the D-loop region, including conserved sequence blocks (CSB) **I-III**, and extended termination associated sequences (ETAS) 1 and 2. Representative BigWig plots of LSP-strand sequences for each concentration are aligned below. All bar graph data are mean normalized to untreated sample values +/- SEM (n=3; p-values calculated by one-way ANOVA: *<0.05, **<0.01, ***<0.001).

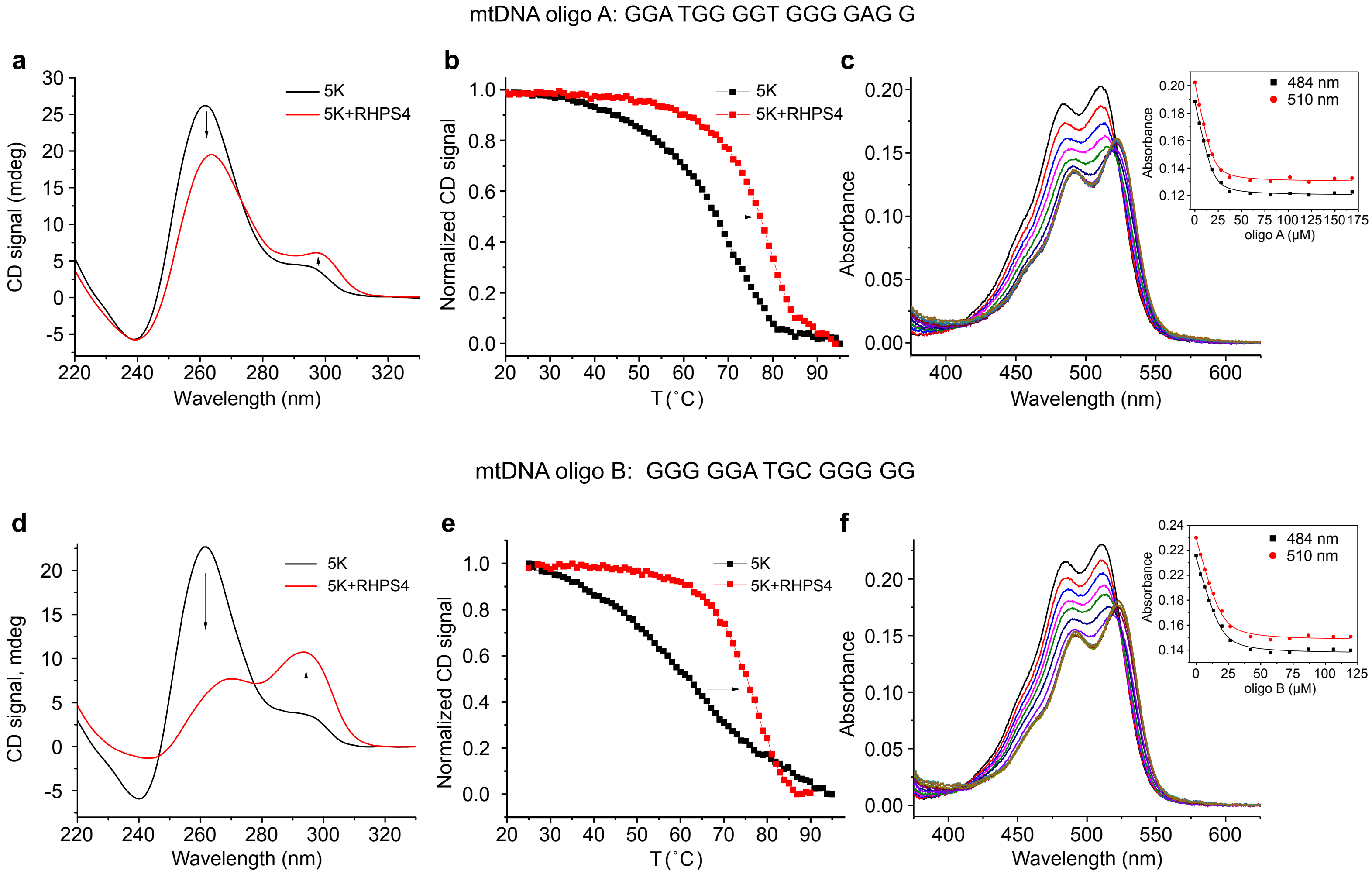

Supplementary Figure S9. RHPS4 stabilizes antiparallel structures in G4-forming mtDNA sequences in oligo A and B. (a,d) CD spectra of 5 μM oligo A (a) or oligo B (d) in the presence (red lines) or absence (black lines) of 10 μM RHPS4. Peak at ~260 nm and ~295 nm indicate parallel and antiparallel content of the folded oligo, respectively. Arrows indicate direction of change upon RHPS4 addition. (b,e) CD melting curves for oligo A (b) or oligo B (e) with and without RHPS4. For oligo A (5 μM), CD signal was monitored at 264 nm with or without RHPS4. For oligo B (5 μM), CD signal was monitored at 260 nm for oligo alone or 295 nm in the presence of RHPS4. Arrow indicates the increase of melting temperature upon RHPS4 treatment. (c,f) RHPS4 absorption profile changes in response to DNA titration. (insert c,f) Direct fit of DNA titration data at specified RHPS4 absorption wavelengths. (c) Binding constant of oligo A was determined to be (0.5 ± 0.3)×106/M with a binding ratio of 3:1 RHPS4-to-G4. (f) Binding constant of oligo B was determined to be (0.5 ± 0.3)×106/M with a binding ratio of 2:1 RHPS4-to-G4.

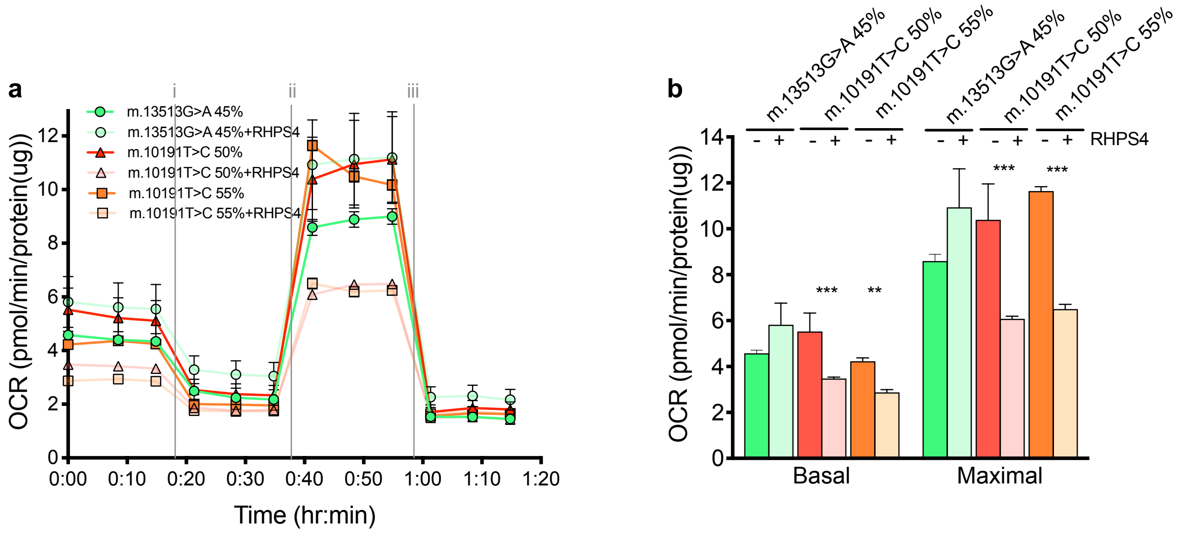

Supplementary Figure S10. Cells harboring a mtDNA mutation that increases the G4 thermal stability and antiparallel characteristics show oxygen consumption defects upon RHPS4 exposure. (a) Example Seahorse XFe96 oxygen consumption rate (OCR) profile for distinct patient fibroblasts harboring 45% m.13513G>A, 50% m.10191T>C, or 55% m.10191T>C mutant mtDNA cultivated for 24 h in presence or absence of 1 µM RHPS4 (n=8 well/line/RHPS4 condition). Treatments were as follows: i) oligomycin; ii) dinitrophenol; and iii) rotenone. Cell number was normalized by protein determination to eliminate differences in growth rate among the different lines. No wells were excluded from analysis. (b) Effects of RHPS4 exposure on basal and maximal oxygen consumption relative to control. All bar graph data are mean +/- SEM (p-values calculated by one-way ANOVA with Dunn's posthoc analysis to test for RHPS4 effects: **<0.01;***<0.001).
